# Biochemistry shapes growth kinetics of nitrifiers and defines their activity under specific environmental conditions

**DOI:** 10.1002/bit.28045

**Published:** 2022-02-11

**Authors:** Eloi Martinez‐Rabert, Cindy J. Smith, William T. Sloan, Rebeca González‐Cabaleiro

**Affiliations:** ^1^ James Watt School of Engineering, Infrastructure and Environment Research Division University of Glasgow Rankine Building Glasgow UK; ^2^ Department of Biotechnology Delft University of Technology Delft The Netherlands

**Keywords:** environmental engineering, kinetic parameters, microbial interaction, nitrifiers

## Abstract

Is it possible to find trends between the parameters that define microbial growth to help us explain the vast microbial diversity? Through an extensive database of kinetic parameters of nitrifiers, we analyzed if the dominance of specific populations of nitrifiers could be predicted and explained. We concluded that, in general, higher growth yield (Y_XS_) and ammonia affinity (a^0^_NH3_) and lower growth rate (µ_max_) are observed for ammonia‐oxidizing archaea (AOA) than bacteria (AOB), which would explain their considered dominance in oligotrophic environments. However, comammox (CMX), with the maximum energy harvest per mole of ammonia, and some AOB, have higher a^0^_NH3_ and lower µ_max_ than some AOA. Although we were able to correlate the presence of specific terminal oxidases with observed oxygen affinities (a^0^_O2_) for nitrite‐oxidizing bacteria (NOB), that correlation was not observed for AOB. Moreover, the presumed dominance of AOB over NOB in O_2_‐limiting environments is discussed. Additionally, lower statistical variance of a^0^_O2_ values than for ammonia and nitrite affinities was observed, suggesting nitrogen limitation as a stronger selective pressure. Overall, specific growth strategies within nitrifying groups were not identified through the reported kinetic parameters, which might suggest that mostly, fundamental differences in biochemistry are responsible for underlying kinetic parameters.

## Graphical abstract

An extensive analysis of kinetic parameter that define microbial growth was developed to understand better the relationships of competition and collaboration of the main contributors of aerobic nitrification. From this analysis, Martinez‐Rabert and co‐workers show that specific metabolic strategies enabling the survival of different populations and also a relationship between biochemical differences and measured growth kinetic parameters. Moreover, it explains our inability to fully describe ecological niche differentiation between different populations involved in the aerobic biogeochemical nitrogen cycle.

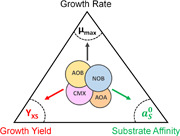

## 1 INTRODUCTION

Advances in culture‐independent studies and metagenomics have greatly increased our knowledge of nitrifying communities revealing that the interactions of this microbial group are not as simple as was once thought (Hugenholtz et al., 1998; Marco, 2011). The nitrification process was traditionally described as a two‐step process of metabolic collaboration between two different populations. Ammonia was always considered to be oxidized first to nitrite by ammonia‐oxidizing bacteria (AOB) and then, nitrite oxidized to nitrate by nitrite‐oxidizing bacteria (NOB). However in 2005 our understanding began to change when archaea oxidizing ammonia to nitrite (ammonia‐oxidizing archaea, AOA) were observed (Könneke et al., 2005; Treusch et al., 2005). Then, in 2015, some NOB populations (*Nitrospira* genus) were proven to fully catalyze the complete ammonia oxidation process to nitrate (named comammox bacteria, CMX) (Daims et al., 2015; van Kessel et al., 2015). Together, with further observations of diverse NOB metabolic activity and new NOB isolates (Daims et al., 2016), the previously underestimated NOB group revealed wide metabolic and physiological diversity. Considering this new information on the complex interspecies relationships of competition and collaboration among populations of nitrifiers, our full understanding of nitrifiers within natural and engineered systems is further challenged. Exploring and understanding the relationship between populations, opportunities for novel designs of biotechnologies might arise, enabling the control of nitrogen concentration in water in a more sustainable way.

Because AOA has an overall higher affinity for ammonia and oxygen than AOB, it is generally considered the dominant population in low ammonia and low pH conditions, and soil and aquatic environments (Baolan et al., 2014; Liu et al., 2017; Yin et al., 2018). AOB in contrary, are generally faster growers than AOA domina in environments where substrate limitation is not the main selective pressure (e.g., wastewater treatment plants) (Lehtovirta‐morley, 2018; Li et al., 2016; Park et al., 2006; Yin et al., 2018). These observations however, have not been fully proven and in many low ammonia environments (<15 µM), such as estuaries or riverine sediments, AOB outnumber AOA (Lagostina et al., 2015; Mosier & Francis, 2008; Santoro et al., 2008). Therefore, although some general conclusions have been established, the relative abundances of both groups of ammonia oxidizers dominating in specific environmental niches remain unknown along with their relative contribution to the global nitrification process.

The few measurements of ammonia affinity for complete nitrification by a single organism (from *Nitrospira inopinata* and Ca. *N. kreftii*), proved to be one of the highest of all affinities reported for ammonia oxidizers (only AOA species *Nitrosopumilus maritimus* and *Nitrosoarchaeum koreensis* have a higher affinity (Jung et al., 2011, 2021; Kits et al., 2017; Sakoula et al., 2020)). With a higher ammonia affinity than AOB, and a more energetic catabolic process per mole of NH_3_ (complete nitrification would yield more energy, ∆G^0'^ = −349 kJ per mole of NH_3_) than single step (∆G^0'^ = −275 kJ per mole of NH_3_ for ammonia oxidation to nitrite, and ∆G^0'^ = −74 kJ per mole of NO_2_ for nitrite oxidation to nitrate) (Daims et al., 2015), CMX would be expected to dominate in oligotrophic environments were substrate availability is limited (Costa et al., 2006). However, CMX have been also identified in a range of engineered systems, including aquaculture biofiltration units, drinking water and wastewater treatment plants (Chao et al., 2016; Pjevac et al., 2017; Wang et al., 2017), with the contribution of their activity to nitrification and their distribution in aforementioned systems still not well understood (Yang et al., 2020). Moreover, the niches in which other populations of NOB dominate are not fully identified, with their lineages unequally distributed in both natural and engineered environments. Some specific NOB species are considered habitat specialists. In particular, *Nitrospina* and *Ca*. Nitromaritima species have been only identified in marine and hypersaline environments, like deep‐sea waters, ocean sediments, and marine oxygen minimum zones (Bristow et al., 2016; Ngugi et al., 2016; Sun et al., 2019), and *Nitrospira* and *Nitrotoga* are usually the dominant NOB in wastewater treatment systems (Daims et al., 2001; Juretschko et al., 1998; Kruse et al., 2013; Lucker et al., 2015). However, the ubiquity of NOB, which reflects their capacity to adapt to several environments, should be a consequence of their intrinsic metabolic diversity (Daims et al., 2016).

The characteristics of specific microbial activities can be associated with identified “*life strategies*.” One such theory is the commonly accepted r/K‐strategy. Those microorganisms that grow fast and dominate in un‐limited substrate environments, such as wastewater treatment systems or eutrophic environments are identified as r‐strategists, with a higher maximum specific growth rate (µ_max_), whereas those microorganisms which grow slowly and dominate in oligotrophic environments are identified as K‐strategists, with higher substrate affinity. A trade‐off between oligotrophic and copiotrophic activity is defined by the r/K‐strategy theory (Andrews & Harris, 1986; Ho et al., 2017).

Thermodynamics and microbial metabolic studies have led us to consider the apparent existence of another trade‐off in kinetic parameters between growth rate and growth yield. This trade‐off would also define theoretical environment strategists, that is, microorganisms defined by a high growth rate and a low growth yield (r‐strategist) versus those with a low growth rate and high growth yield (Y‐strategist) (Kreft, 2004; Pfeiffer et al., 2001). This trade‐off is supported by the measurement of a constant rate of metabolic redox activity, which implies that longer metabolic pathways will potentially harvest more energy but require more time to metabolize one mole of substrate (Andersen & Von Meyenburg, 1980; González‐Cabaleiro et al., 2015; Hoff et al., 2020). The branched metabolic pathways of *Escherichia coli*, *Holophaga foetida*, and *Acetobacter methanolicus* (Carlson & Srienc, 2004; Kappler et al., 1997; Müller & Babel, 1993); or the competition between fermentative pathways of C*lostridium homopropionicum* (r‐strategist) and *Propionibacterium freudenreichii* (Y‐strategist) (Seeliger et al., 2002) support the existence of growth rate/yield trade‐off.

These theories further identify that no microorganism can be a “*Jack of all trades*”, but there is not an understanding on what at the molecular mechanistic level defines a microorganism as r‐ or K‐ or Y‐strategist. Moreover, the fitness of specific microbial species is not strictly fixed, but able to adapt to dynamic environmental conditions (Velicer & Lenski, 1999).

In this study, we analyzed the kinetic parameters of AOB, AOA, CMX, and NOB, reviewing approximately 100 references in literature and more than 300 data points, with the objective to understand better the relationships of competition and collaboration established between different functional groups of aerobic nitrifiers. With it, we aim to predict the ecological niches in which specific populations of nitrifiers will dominate. Values of maximum specific growth rate (µ_max_), growth yield (Y_XS_), and the affinities for oxygen and nitrogen sources (a^0^_O2_ and a^0^_N_), were collected, normalized, and compared for each of the potential groups competing for the same substrate. The analysis of the data highlights the specific metabolic strategies enabling the survival of different populations, and the relationship between biochemical differences and measured kinetic parameters. Moreover, it explains our inability to fully describe ecological niche differentiation between the populations involved in the aerobic biogeochemical nitrogen cycle.

## 2 MATERIALS AND METHODS

In this study, a database of the kinetic parameters for nitrifiers reported in the literature was collated. Maximum specific growth rate (µ_max_), apparent growth yield (Y_XS_), and specific affinity for ammonia (a^0^_NH3_), oxygen (a^0^_O2_), and nitrite (a^0^_NO2_) have been annotated and compared for different aerobic nitrifying groups. To enable the comparison, the following extrapolations and conversions were done:

### 2.1 Maximum specific growth rate (µ_max_)

Maximum specific growth rate is presented in this study in units of h^−1^ at a constant temperature of 20°C for all the measurements. To do this, when necessary, the values obtained from literature were extrapolated to 20°C using an Arrhenius function (Equation 1) (Melcer, 2004).

(1) $$\mu_{T1}=\mu_{T2}\times \theta^{T1-T2}.$$

In Equation (1), θ refers to the dimensionless Arrhenius coefficient. Linear regression and least squares method were applied to fit an Arrhenius function to the experimental data for each µ_max_ value collected from the literature. A table with the values is presented in Supplementary Online Materials (Tables S1 and S2).

To normalize the effect of pH, all values were extrapolated at the pH considered optimum for each species or genus. All optimum pH values are reported between 7 and 8 for the nitrifying groups considered (Figure S1). To extrapolate the µ_max_ value at its optimum pH, a function with a bell curve shape was used to define the effect of pH over the µ_max_ values (Equation 2) (Antoniou et al., 1990; Blackburne et al., 2007a, 2007b; Dochain & Vanrolleghem, 2015; French et al., 2012; Jung et al., 2011; Kitzinger et al., 2018; Lafuente et al., 2008; Qin et al., 2014; Tourna et al., 2011).

(2) $$\mu_{max}\left(\mathrm{pH},)\right)=\frac{\mu_{max}\left({\mathrm{pH}}_{\mathrm{op}}\right)}{1+\left(\frac{10^{-pK1}}{10^{-\mathrm{pH}}}\right)+\left(\frac{10^{-\mathrm{pH}}}{10^{-pK2}}\right)}.$$

In Equation (2), pK_1_ and pK_2_ refer to the pH in which µ_max_ is half of the value at optimal pH (see Supplementary Online Materials).

### 2.2 Specific affinities for substrates (a^0^_NH3_, a^0^_NO2_, and a^0^_O2_)

Specific affinity (a^0^) evaluates the capacity of microorganisms to survive under specific substrate concentrations (Button, 1991). Specific affinities for ammonia, nitrite, and oxygen were calculated using the data of kinetic constants form literature for AOB, AOA, CMX, and NOB and applying Equation (3) (Button, 1985).

(3) $$a_{S}^{0}=\frac{\left(V_{\max}\right)}{K_{M}}.$$

Here $a_{S}^{0}$ is the specific affinity for *S* $\left(L\;g‐{\mathrm{Bio}}^{-1}\;h^{-1},)\right)$, $V_{\max}$ is maximum specific uptake rate $\left(µ\text{mol}‐S\;{g‐\text{Bio}}^{-1}h^{-1}\right)$ and K_M_ is half‐saturation constant for *S* (µM). The literature data are included in Tables S3–S5.

### 2.3 Growth yield (Y_XS_)

Growth yield or apparent growth yield is defined as the amount of biomass produced per unit of substrate consumed, considering that part of the substrate consumed is required for the maintenance processes. We present the apparent growth yield in units of gBio/gNH_3_ for ammonia oxidizers and gBio/gNO_2_^−^ for nitrite oxidizers. To transform the reported growth yield to these units when needed, an average formula for biomass was considered (C_5_H_7_O_2_N). Other conversion factors used are included in Tables S6 and S7.

### 2.4 Statistical analyses

Statistical significance of the differences between the parameters describing growth (maximum specific growth rate (µ_max_), growth yield (Y_XS_), and specific affinity (a^0^)) of the nitrifying groups considered (AOB, AOA, CMX, and NOB) was assessed using the one‐way ANOVA analysis together with REGWQ TEST. To evaluate the correlations between maximum specific growth rate (µ_max_), growth yield (Y_XS_), and specific affinity (a^0^) Pearson's correlation coefficient (*r*) was used.

## 3 RESULTS AND DISCUSSION

The collected kinetic parameters of ammonia and nitrite oxidizers were organized in groups based on their metabolic activity, domain, and origin (Tables 1 and 2) function of available taxonomic information (genus and species). Then, the values were classified into seven different ecological groups as a function of the microorganism and its habitat: non‐marine ammonia‐oxidizing bacteria (AOB‐FW), marine ammonia‐oxidizing bacteria (AOB‐SW), nonmarine ammonia‐oxidizing archaea (AOA‐FW), marine ammonia‐oxidizing archaea (AOA‐SW), comammox bacteria (CMX), non‐marine nitrite‐oxidizing bacteria (NOB‐FW), and marine nitrite‐oxidizing bacteria (NOB‐SW). The groups are also distinguished by the ecosystem they were isolated from: wastewater treatment systems, sediments (including oceanic, estuarine, and lake sediments), water column, soils, hot water/spring, and acidic soils.

**Table 1:** Summary of the kinetic parameters of ammonia oxidizers included in the database used in this study

	Abbreviation	Taxonomic level and culture type^a^	Parameters	Ecosystem^b^
Non‐marine ammonia‐oxidizing bacteria (AOB‐FW)				
Mixed culture	Mx AOB‐FW	Mixed culture	All^c^	WWTP
* Nitrosomonas europaea*	Europaea	Species, PC	All	Soil
* Nitrosomonas oligotropha*	Oligotropha	Species, PC and EC	µ_max_, a^0^_NH3_	Sediments
* Nitrosospira sp*. 40K1	Nspira‐40K1	Species, PC	µ_max_, a^0^_NH3_, Y_XS_	Soil
* Nitrosospira sp*. AF	Nspira‐AF	Species, PC	µ_max_, a^0^_NH3_, Y_XS_	Acidic soil
* Nitrosospira sp*. B6	Nspira‐B6	Species, PC	µ_max_, a^0^_NH3_, Y_XS_	WWTP
* Nitrosospira sp*. L115	Nspira‐L115	Species, PC	µ_max_, a^0^_NH3_, Y_XS_	Acidic soil
Marine ammonia‐oxidizing bacteria (AOB‐SW)				
* Nitrosococcus oceani*	Oceani	Species, PC	µ_max_, a^0^_NH3_, Y_XS_	Sediments
Non‐marine ammonia‐oxidizing archaea (AOA‐FW)				
* Nitrosoarchaeum koreensis*	Koreensis	Species, EC	µ_max_, a^0^_NH3_, a^0^_O2_	Soil
* Nitrososphaera vienennsis*	Vienennsis	Species, PC	µ_max_, a^0^_NH3_, Y_XS_	Soil
* Nitrososphaera gargensis*	Gargensis	Species, PC	µ_max_, a^0^_NH3_, Y_XS_	Hot spring
Marine ammonia‐oxidizing archaea (AOA‐SW)				
Mixed culture	Mx AOA‐SW	Mixed culture	µ_max_, a^0^_O2_	Sediments
* Nitrosopumilus maritimus*	Maritimus	Species, PC	All	Sediments
* Nitrosopumilus piranensis*	Piranensis	Species, EC	µ_max_, Y_XS_	Water column
* Nitrosopumilus adiactus*	Adriaticus	Species, EC	µ_max_, Y_XS_	Water column
Complete ammonia‐oxidizing bacteria (CMX)				
* Nitrospira inopinata*	Inopinata	Species, PC	µ_max_, a^0^_NH3_, Y_XS_	Hot water

^a^ Culture type: PC – pure culture; EC – enriched culture.

^b^ Ecosystem (sample origin): WWTP – Wastewater treatment plants.

^c^ All: All microbial growth parameters have been reported, µ_max_, a^0^_NH3_, a^0^_O2_, and Y_XS_.

**Table 2:** Summary of the kinetic parameters of nitrite oxidizers included in the database used in this study

	Abbreviation	Taxonomic level and culture type^a^	Parameters	Ecosystem^b^
Non‐marine nitrite‐oxidizing bacteria (NOB‐FW)				
* Nitrobacter vulgaris*	Vulgaris	Species, PC	µ_max_, a^0^_NO2_, Y_XS_	WWTP
* Nitrospira sp. ND1*	ND1	Species, PC	All^c^	WWTP
* Nitrospira japonica*	Japonica	Species, PC	All	WWTP
* Nitrobacter agilis*	Agilis	Species, PC	µ_max_, a^0^_NO2_, Y_XS_	WWTP
* Nitrobacter winogradskyi*	Winogradsky	Species, PC	All	Soil
* Nitrospira defluvii*	Defluvii	Species, PC	µ_max_, a^0^_NO2_,Y_XS_	WWTP
* Nitrospira lenta*	Lenta	Species, PC	µ_max_, a^0^_NO2_,Y_XS_	WWTP
* Nitrospira moscoviensis*	Moscoviensis	Species, PC	µ_max_, a^0^_NO2_,Y_XS_	Hot water
* Nitrobacter hamburgensis*	Hamburgensis	Species, PC	All	Soil
* Nitrotoga arctica*	Arctica	Species, PC	µ_max_, a^0^_NO2_,Y_XS_	Soil
Marine nitrite‐oxidizing bacteria (NOB‐SW)				
* Nitrococcus mobilis*	Mobilis	Species, PC	µ_max_, a^0^_NO2_	Water column
* Nitrospira marina*	Marina	Species, PC	µ_max_, Y_XS_	Water column
* Nitrospina watsonii*	Watsonii	Species, EC	µ_max_, a^0^_NO2_,Y_XS_	Water column
* Nitrotoga* sp. AM1	AM1	Species, EC	µ_max_, a^0^_NO2_	Sediments
* Nitrospira* sp. Ecomares	Ecomares	Species, PC	µ_max_, a^0^_NO2_,Y_XS_	Sediments

^a^ Culture type: PC – pure culture; EC – enriched culture.

^b^ Ecosystem (sample origin): WWTP – Wastewater treatment plants.

^c^ All: All microbial growth parameters have been reported, µ_max_, a^0^_NO2_, a^0^_O2_, and Y_XS_.

**Figure 1:**
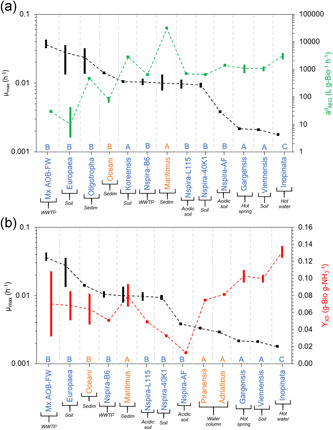
Maximum specific growth rate (µ_max_) with (a) specific affinity for ammonia (a^0^_NH3_) and (b) growth yield (Y_XS_) of ammonia‐oxidizing microorganisms (AOB, AOA, and CMX). The black bars show the range of µ_max_ values; green bars represent the range of a^0^_NH3_ value for ammonia (a); and red bars represent the range of Y_XS_ values (b). Blue: non‐marine nitrifiers; orange: marine nitrifiers. Legend bottom of figures: B – Bacteria; A – Archaea; C – Complete ammonia oxidizer. Dashed lines cross the calculated average value for each parameter function of the range of values reported

**Figure 2:**
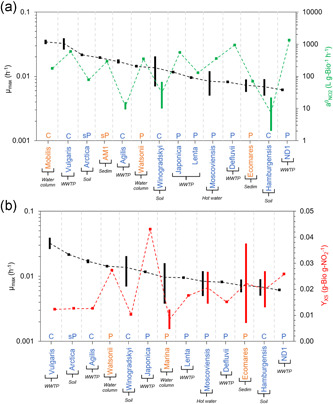
Maximum specific growth rate (µ_max_) with (a) specific affinity for nitrite (a^0^_NO2_) and (b) growth yield (Y_XS_) of nitrite‐oxidizing bacteria. The black bars show the range of µ_max_ values; green bars represent the range of a^0^_NO2_ values (a); red bars represent the range of Y_XS_ value (b). Blue: non‐marine nitrite oxidizers; orange: marine nitrite oxidizers. Legend bottom of figures: C – NOB with cytoplasmic NXR; P – NOB with periplasmic NXR. sP – NOB with soluble periplasmic NXR. Dashed lines cross the calculated average value for each parameter function of the range of values reported

**Figure 3:**
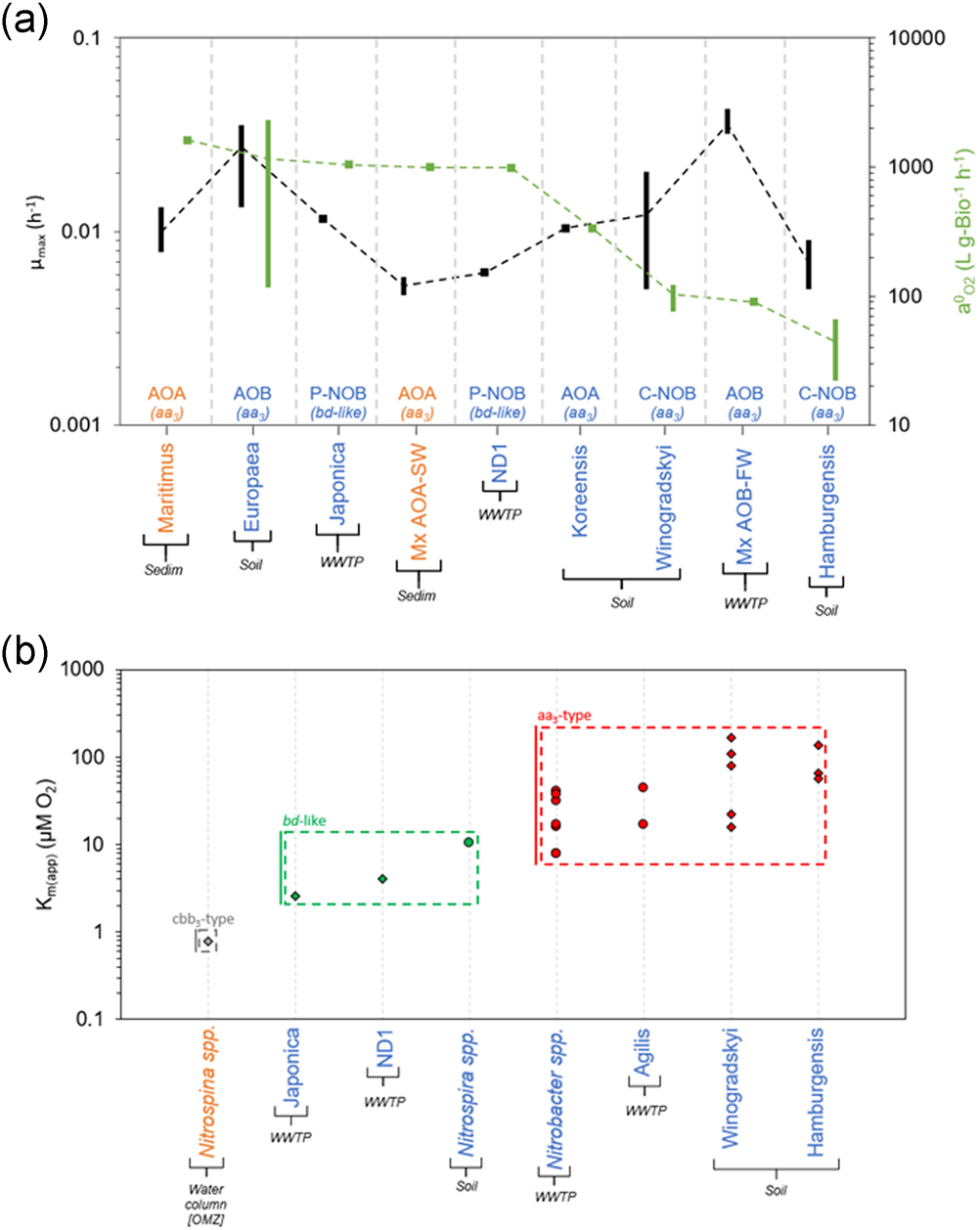
(a) Maximum specific growth rate (µ_max_) with specific affinity for oxygen (a^0^_O2_) for all the nitrifiers’ populations considered. The black bars show the range of µ_max_ values; dark green bars represent the range of a^0^_O2_ values; and dashed connect the average of each value range. Blue: marine nitrifiers; orange: non‐marine nitrifiers. Legend bottom of figures: AOB – Ammonia‐oxidizing bacteria; AOA – Ammonia‐oxidizing archaea; P‐NOB – NOB with periplasmic NXR; C – NOB with cytoplasmic NXR. On the bottom of tags, in parentheses, the terminal oxidase that each group uses to reduce oxygen is shown (Table S8). Dashed lines cross the calculated average value for each parameter function of the range of values reported. (b) Apparent substrate affinity (K_m(app)_) for oxygen of NOB. K_m(app)_ values are given for growth measurements (circles) and activity measurements (diamonds). Marker color legend – Red: NOB with heme‐copper oxidase aa3‐type as terminal oxidase; Green – NOB with putative cytochrome bd‐like oxidase as terminal oxidase; Gray – NOB with heme‐copper oxidase cbb3‐type as terminal oxidase. [OMZ]: samples from oxygen minimum zones (OMZ). K_m(app)_ of Nitrospinae is significantly different from that of Nitrospira and Nitrobacter species (*p* < 0.0001) and K_m(app)_ of Nitrospira species are significantly different from that of Nitrobacter species (*p* < 0.0001). See Table S8 for references about inventory of terminal oxidase of NOB. See Table S10 for references of K_m(app)_ values

The maximum specific growth rate (µ_max_) of AOB, AOA, and CMX is compared with the specific affinity for ammonia (a^0^_NH3_) (Figure 1a) and with the growth yield (Y_XS_) (Figure 1b). For NOB, the µ_max_ values are plotted with the specific affinities for nitrite (a^0^_NO2_) (Figure 2a) and growth yield (Y_XS_) (Figure 2b). For all nitrifying groups, the specific affinities for oxygen (a^0^_O2_) are presented in Figure 3a with their µ_max_. Data shown in Figures 1 and 2 have been organized from the highest to the lowest maximum specific growth rate. Data shown in Figure 3 has been organized from the highest to the lowest affinity for oxygen.

### 3.1 Ammonia oxidizers

Collected data of ammonia oxidizers (Figure 1a) shows that AOB populations have on average a higher maximum specific growth rate than AOA and CMX (0.021 ± 0.012 h^−1^ (*n* = 20) for AOB, 0.006 ± 0.004 h^−1^ (*n* = 7) for AOA and 0.002 h^−1^ (*n* = 1) for CMX). But AOA and CMX have on average a higher specific affinity for ammonia than AOB (4242.89 ± 9461.33 L·g‐Bio^−1^·h^−1^ (*n* = 10) for AOA, 4287.66 ± 1765.09 L·g‐Bio^−1^·h^−1^ (*n* = 2) for CMX and 240.00 ± 390.75 L·g‐Bio^−1^·h^−1^ (*n* = 17) for AOB). The available measurements of the kinetics of complete nitrifiers show they have the lowest maximum specific growth rate (being close to some µ_max_ values reported for AOA) and the highest affinity for ammonia of all analyzed ammonia oxidizers except *N. maritimus* and *N. koreensis*. This overall tendency would confirm the consideration of AOA and CMX as K‐strategists when compared with AOB, with lower µ_max_ and higher ammonia affinity (Chen et al., 2017; Yin et al., 2018). When analyzing the reported values of µ_max_ and a^0^_NH3_ in literature for AOB, AOA, and CMX groups (Figure 1a), we identify a strong negative correlation (*r* = −0.717; *p* < 0.006; *n* = 13, Figure S2a), supporting the aforementioned consideration that AOA and CMX have higher a^0^_NH3_ and lower µ_max_. A negative correlation is also observed between the data collected for AOB populations only (*r* = –0.808; *p* = 0.015; *n* = 8, Figure S2b) but we found a strong positive correlation between the µ_max_ and a^0^_NH3_ values for populations of AOA (*r* = 0.756; *p* = 0.02; *n* = 4, Figure S2c). Then, although we are able to identify some species of AOB that will preferentially dominate in oligotrophic environments, and this supports the r/K‐strategy theory (*Nitrosomonas* have consistently higher µ_max_ and lower a^0^_NH3_ than *Nitrosococcus* or *Nitrosospira*), we have not been able to find a similar trend between populations of AOA.

It is important to consider that AOA was the only cohort identified in extreme oligotrophic environments such as the oxygen minimum zones (OMZ) (Bristow et al., 2016). This excellent capacity of AOA to survive in these extreme environments is observed, for example, on the measured a^0^_NH3_ of *N. maritimus*, which is 22 times higher than the highest measured a^0^_NH3_ of AOB. However, in some natural environments identified as oligotrophic environments, AOB outcompeted AOA (Lagostina et al., 2015; Mosier & Francis, 2008; Santoro et al., 2008). This correlates with the measured a^0^_NH3_ shown in Figure 1a. *Nitrosospira* species have a similar a^0^_NH3_ than some AOA species (Figures 1a and S5) being able to compete against some AOA in these oligotrophic environments.

In Figure 1b, µ_max_ is compared with the growth yield (Y_XS_) of each ammonia oxidizer considered. As expected, complete nitrifiers show the highest Y_XS_ value (Kits et al., 2017), but also there is a significant difference between the reported Y_XS_ of AOB and AOA, both groups carrying out partial nitrification (0.054 ± 0.024gBio/gNH_3_ (*n* = 9) and 0.088 ± 0.014gBio/gNH_3_ (*n* = 9), respectively; *p* = 0.002). AOA has a consistently higher Y_XS_ than AOB, consequence of a more efficient metabolism. The carbon fixation pathway of AOA has been reported as more efficient (3‐hydroxypropionate/4‐hydroxybutyrate (HP/HB) cycle) than the Calvin–Benson–Bassham cycle of AOB (Könneke et al., 2014).

When analyzing the reported values for µ_max_ and Y_XS_ for AOB (excluding acidophilic AOB: *Nitrosospira sp. AF* and *Nitrosospira sp. L115*), AOA and CMX, we identify a weak negative correlation (*r* = –0.404; *p* < 0.1; *n* = 11, Figure S2d), which supports the hypothesis of an inverse correlation between metabolic efficiency and speed of growth (Kreft, 2004; Lele & Watve, 2014). A negative correlation is also observed between the parameters reported for AOA (*r* = −0.506; *p* = 0.002; *n* = 5, Figure S2e), but not for AOB (*r* = 0.808; *p* = 0.05; *n* = 6, Figure S2f).

In addition, non‐marine AOA have a higher average value of Y_XS_ than marine AOA (0.100 ± 0.007gBio/gNH_3_ (*n* = 3) for AOA‐FW and 0.078 ± 0.009gBio/gNH_3_ (*n* = 5) for AOA‐SW; *p* = 0.01) (Figure 1b). This higher value of Y_XS_ is also associated with lower µ_max_ values. Contrary, this difference in metabolic efficiency is not observed when non‐marine and marine AOB are compared (*p* > 0.1; Figure S4). Regarding acidophilic AOB, we observe a significantly lower values of Y_XS_ in comparison to neutrophilic AOB (*p* < 0.01; Figure S4). These dissimilarities could be a consequence of the significantly different maintenance requirements of the different environments (Bodegom, 2007). In fact, no trend has been identified between µ_max_ and Y_XS_ parameters within the same ecological group.

Overall, for ammonia oxidizers we have identified negative correlations between maximum growth rate and ammonia affinity and growth yield, respectively. Therefore, microorganisms that have higher growth yield tend to have higher ammonia affinity meanwhile being slow growers in conditions of non‐substrate limitation. In general, we observe lower µ_max_, higher a^0^_NH3_, and higher Y_XS_ for AOA and CMX than for AOB, which indicates that these groups have a competitive advantage in substrate limiting conditions.

### 3.2 Nitrite oxidizers

In addition to the main groups (NOB‐FW and NOB‐SW), species of NOB are classified based on the localization of the active site of their nitrite oxidoreductase (NXR), the enzyme catalyzing nitrite oxidation to nitrate, differentiating between cytoplasmic NXR (C‐type NOB), periplasmic NXR (P‐type NOB), and soluble periplasmic NXR (sP‐type NOB). In general, *Nitrobacter* and *Nitrococcus* are C‐type NOB, *Nitrospira* and *Nitrospina* are P‐type NOB and *Nitrotoga* are sP‐type NOB (Füssel et al., 2017; Koch et al., 2015; Lücker et al., 2010; Lücker et al., 2013; Spieck et al., 1996; Spieck et al., 1998; Starkenburg et al., 2006).

Figure 2a shows that C‐type NOB have a significantly lower affinity for nitrite (a^0^_NO2_) than P‐type NOB (*p* < 0.0001) and sP‐type NOB (*p* < 0.0001) (74.17 ± 168.81 L·g‐Bio^−1^·h^−1^ (*n* = 23) for C‐type NOB, 527.28 ± 451.01 L·g‐Bio^−1^·h^−1^ (*n* = 7) for P‐type NOB and 145.76 ± 91.49 L·g‐Bio^−1^·h^‐1^ (*n* = 5) for sP^‐^type NOB). However, no correlation has been found between µ_max_ and Y_XS_ parameters (Figure 2b). Commonly, *Nitrobacter* (C‐type NOB) are considered r‐strategists and *Nitrospira* (P‐type NOB) are considered K‐strategists (Nowka et al., 2015; Schramm et al., 1999). However, this is not supported by the present analysis as no correlation between µ_max_ and a^0^_NO2_ is found (*r* = 0.062; *p* > 0.1; *n* = 14, Figure S2g). Analyzing the kinetic data shown in Figure 2b, we identify a weak negative correlation between µ_max_ and Y_XS_ for NOB (*r* = –0.29; *p* > 0.1; *n* = 13, Figure S2h). Suggested location of Figure 2.

P‐type NOB release protons in the periplasmic side of the membrane as nitrite oxidation occurs. This could imply the generation of an extra unit of proton motive force. It has been therefore considered that P‐type NOB would have a more efficient metabolism than C‐type (Lücker et al., 2010). Contrary, no significant difference between reported Y_XS_ values for P‐type NOB and C‐type NOB has been observed (0.021 ± 0.012gBio/gNO_2_ (*n* = 11) for P‐type NOB and 0.022 ± 0.012gBio/gNO_2_ (*n* = 10) for C‐type NOB; *p* = 0.73). Other morphological differences might be affecting the efficiency of the metabolic process, for example, the distinct terminal oxidoreductases that they express or the different carbon fixation pathways of *Nitrobacter* (Calvin–Benson–Bassham cycle, CBB) and *Nitrospira* (oxygen tolerant modified *reductive tricarboxylic acid cycle*, rTCA) (Lücker et al., 2010; Lücker et al., 2013; Starkenburg et al., 2006; Starkenburg et al., 2008). Although it is established that rTCA is more efficient than CCB (0.195 moles ATP per g biomass and 0.238 moles ATP per g biomass respectively) (Berg, 2011; Mangiapia & Scott, 2016), this is not reflected in the measured growth yields of NOB (Berg, 2011; Sato et al., 2014). Moreover, *Nitrobacter* encode a heme‐copper aa_3_‐type as terminal oxidase that operates as proton pump, whereas *Nitrospira* encode a putative cytochrome *bd*‐like terminal oxidase (Table S8) that could not be coupled with energy conservation (or can conserve energy via a Q‐loop, but less than a proton‐pumping mechanism), like the canonical *bd* terminal oxidase (Giuffre et al., 2014). This might compensate the putative energetic advantage of *Nitrospira* by the orientation of their NXR and carbon fixation pathway.

As observed in the analysis of the kinetic parameters of AOB, there are no significant differences between Y_XS_ values when marine and non‐marine NOB are compared (*p* > 0.1; Figure S7). Regarding to µ_max_ and a^0^_NO2_ values for NOB populations, we observed a significant variation between *Nitrobacter, Nitrococcus*, and *Nitrotoga* species from distinct environments (*p* < 0.0001), but there is less variation between those of *Nitrospira* and *Nitrospina* species (Figures S6 and S8).

### 3.3 Oxygen competition among nitrifiers

Oxygen is the main electron acceptor for nitrification, and therefore the seven ecological groups compete for it. Figure 3a presents the specific affinity for oxygen (a^0^_O2_) for all nitrifying groups considered except CMX (their a^0^_O2_ has not been reported yet) (Figure 3). Suggested location of Figure 3.

No correlation between the µ_max_ and a^0^_O2_ values of considered nitrifying groups was observed (*r* = –0.10; *p* = 0.61; *n* = 9, Figure S2I) (Figure 3a). In addition, diversity in a^0^_O2_ values for all species considered is significantly lower than for the values gathered for a^0^_NH3_, and a^0^_NO2_ (Figures S5, S8, and S9). Between NOB populations, *Nitrobacter* is identified as the group with the lowest affinity for oxygen and *Nitrospira* with the highest. Considering the K_m,(app)_ values for oxygen of NOB (Figure 3b), we could assume that *Nitrospina* genus would have a higher affinity for oxygen than *Nitrospira*. This correlates with the intrinsic K_O2_ values of the terminal oxidases of each of the NOB populations (Tables S8 and S9). Considering only NOB, a positive correlation between affinity of the terminal oxidase of the species considered and the specific affinity measured is observed (Figure 3a,b). However, no correlation between the terminal oxidase and a^0^_O2_ for AOA and AOB groups is observed (Figure 3a). Although, AOA and AOB are reported as carrying an aa_3_‐type terminal oxidase, which is the oxidase with the lowest affinity for oxygen (Table S9), Figure 3a shows that AOA and AOB, except Mx AOB‐FW, have a similar oxygen affinity to *Nitrospira*, which encodes a *bd*‐like terminal oxidase (Table S8). This lack of correlation might be explained by the presence of the monooxygenation step in ammonia oxidation (Arp et al., 2002; Vajrala et al., 2013). This additional oxygen consumption could increase the oxygen concentration gradient between cytoplasm and periplasm and, as consequence, intensify the penetration ratio of oxygen into the cell independiently of the specific affinity of the encoded terminal oxidase (Harder & Dijkhuizen, 1983; Tempest & Neijssel, 1978).

In general, it is considered that AOA has a higher affinity for oxygen than AOB (Liu et al., 2017; Yin et al., 2018). However, the measured affinities for oxygen of AOA and AOB considered in this analysis, show not significant differences, suggesting that AOB populations could compete against AOA even in oxygen limiting conditions (984.16 ± 640.77 L/g‐Bio/h (*n* = 4) for AOA and 1045.90 ± 834.92 L/g‐Bio/h (n = 9) for AOB; *p* = 0.72). On the other hand, AOB populations tend to be considered better competitors for oxygen than NOB (Lafuente et al., 2008; Wiesmann, 1994), but Figure 3a shows that AOB have a significantly higher oxygen affinity than C‐type NOB (1045.90 ± 834.92 L/g‐Bio/h (*n* = 9) for AOB and 171.53 ± 260.28 L/g‐Bio/h (*n = 7*) for C‐type NOB; *P* < *0.005*) and similar affinity values to P‐type NOB (1045.90 ± 834.92 L/g‐Bio/h (*n* = 9) for AOB and 1016.36 ± 41.75 L/g‐Bio/h (*n* = 2) for P‐type NOB; *p* = 0.63). This analysis supports that ammonia oxidizers would only dominate the competition for oxygen if *Nitrobacter* is the dominant population in the NOB community but contrary, NOB would compete closely for oxygen with populations of AOB or AOA if *Nitrospira* are abundant in the NOB community.

## 4 CONCLUSIONS

The present analysis on the kinetics of aerobic nitrifiers, identifies specific trends between the parameters of the different populations in the community. High affinity for a substrate does not guarantee the survival of a microorganism in oligotrophic environments if the catabolic activity at low substrate concentrations does not ensure the harvest of enough energy. Likewise, it might not be competitive to carry an efficient but slower metabolism if essential substrates cannot be assimilated in conditions of low concentrations. Those microorganisms which have evolved to thrive in oligotrophic environments, might tend to be metabolically efficient (high Y_XS_), and show a high substrate affinity (high a^0^). In this study, we have demonstrated that high growth yield correlates with high substrate affinity for those populations of nitrifiers that dominate in environments where substrate limitation is a fundamental selective pressure. Figure 1 shows that in general, AOA and CMX present low µ_max_, high a^0^_NH3_, and high Y_XS_, whereas AOB show higher µ_max_, lower a^0^_NH3_, and lower Y_XS_.

Nevertheless, Figure 1a shows that not all AOA have a significant higher affinity for ammonia than AOB which could explain reported dominance of AOB over AOA, in some natural oligotrophic environments. Also, Figure 3a shows the inconsistence of the assumption that AOB has a higher affinity for oxygen than NOB (although *Nitrobacter* presents a lower affinity for oxygen, *Nitrospira* has a similar affinity than ammonia‐oxidizers). Notably, we observe that for all the groups, the range of values found for a^0^_O2_ is lower than for a^0^_NH3_ or a^0^_NO2_, which can be a reflection of nitrogen availability acting as a stronger selective pressure.

From this comprehensive analysis of the kinetic parameters of nitrifiers, no specific ecological strategies associated with a specific genus or species within the same ecological groups of nitrifiers were identified. Mainly fundamental differences in the biochemistry of the different populations of nitrifiers (e.g., complete vs. partial ammonia oxidation, archaea vs. bacteria, different terminal oxidases, different carbon fixation pathways, or periplasmic vs. cytoplasmic NXR), lead to significant differences in the measured kinetic parameters and potential niche specializations. This msuggests that the kinetics associated with any microbial species might be determined by the specific metabolic traits and activity catalyzed, with constrained capacity for adaptation.

## CONFLICT OF INTERESTS

The authors declare no conflict of interest.

## Supporting information

Supporting information.

## Data Availability

The data that supports the findings of this study are available in the supplementary material of this article.
