# Supplementary material for: Biochemistry shapes growth kinetics of nitrifiers and defines their activity under specific environmental conditions
Source: Biotechnol Bioeng. 2022 Feb 11;119(5):1290–300. doi: 10.1002/bit.28045 (PMC9303882; doi:10.1002/bit.28045)
Supplement: Supplementary file 1 — Supporting information. [file BIT-119-1290-s001.docx]

**Supplementary Information**

**Biochemistry shapes growth kinetics of nitrifiers and defines their activity under specific environmental conditions**

Eloi Martinez-Rabert^a^, Cindy J. Smith^a^, William T. Sloan^a^, Rebeca González-Cabaleiro^b*^

*^a^James Watt School of Engineering, Infrastructure and Environment, University of Glasgow, Rankine Building, Glasgow, G12 8LT, UK*

*^b^Department of Biotechnology, Delft University of Technology, Van der Maasweg 9, 2629 HZ Delft, The Netherlands*

**Correspondence:* Rebeca González-Cabaleiro. Department of Biotechnology, Delft University of Technology, Van der Maasweg 9, 2629 HZ Delft, The Netherlands. E‑mail: *r.gonzalezcabaleiro@tudelft.nl*

**Contents**

Supplementary Text – Materials and Methods

Supplementary Tables – Tables S1-S12

Supplementary Figures – Figures S1-S9

Supplementary References

**Supplementary Text – Materials and Methods**

*Estimation of kinetic parameters. Temperature and pH*

To obtain the values of θ (Equation 1), linear regression and least squares method is applied to set the best fit of the Arrhenius function to the experimental data. First, the Equation 1 is linearized using natural logarithm (Equation S1), considering that θ is the exponential of constant C (*e*^C^).

$\ln\left( \frac{\mu_{T1}}{\mu_{20}} \right)=\theta\cdot(T-20)$ (S1)

Then, a linear regression is applied to obtain an initial value of C and, subsequently, a value of θ. Finally, least squares method is used to set better the equation to the experimental data, using as initial values of θ those obtained from the linear regression. Table S1 presents the values of θ obtained and also the references of the experimental data used to calculate them. Analogously, the parameters that correlated the pH influence with the µ^max^ values, (Equation 2) are obtained by least squares method, assigning the initial values of *pK1* and *pK2* considering their definition: µ_max_(pK1) = µ_max_(pK2) = ½·µ_max_(pH_op_). The kinetic parameters and experimental data used to obtain the equation parameters provided from bibliographic literature searched (see Figure S1).

**Supplementary Tables**

**Table S1.** Arrhenius coefficient (θ) of nitrifying bacteria and archaea. Effect of temperature on maximum growth rate

| **Culture** | **θ** | **Temperature range (ºC)** | **pH** | **References** |
| --- | --- | --- | --- | --- |
| **Ammonia-oxidizing bacteria (non-marine)** | | | | |
| Mixed culture | 1.114 | 7.0 – 21.0 | 7.0 – 7.8 | ^† (1)^ |
| *Nitrosomonas* species | 1.098 | 7.0 – 30.0 | 7.0 – 7.8 | ^† (2)^ |
| *Nitrosospira* species | 1.126 | 3.0 – 21.0 | 7.5 | ^† (3)^ |
| *Nitrosospira sp.* 40K1 | 1.110 | 3.0 – 21.0 | 7.5 | [1] |
| *Nitrosospira sp.* AF | 1.146 | 3.0 – 31.0 | 7.5 | [1] |
| *Nitrosospira sp.* B6 | 1.114 | 3.0 – 26.0 | 7.5 | [1] |
| *Nitrosospira sp.* L115 | 1.132 | 3.0 – 26.0 | 7.5 | [1] |
| **Ammonia-oxidizing bacteria (marine)** | | | | |
| Mixed culture | 1.114 | 7.0 – 21.0 | 7.0 – 7.8 | ^† (1)^ |
| *Nitrosomonas* species | 1.098 | 7.0 – 30.0 | 7.0 – 7.8 | ^† (2)^ |
| **Ammonia-oxidizing archaea (non-marine)** | | | | |
| Mixed culture | 1.126 | 10.0 – 30.0 | 6.0 – 7.5 | ^† (4)^ |
| *Nitrososphaera koreensis* | 1.150 | 15.0 – 30.0 | 6.0 – 8.0 | [2] |
| *Nitrososphaera vienennsis* | 1.180 | 10.0 – 30.0 | ? | [3] |
| *Nitrososphaera gargensis* | 1.126 | 20.0 – 46.0 | ? | ^† (4)^ |
| **Ammonia-oxidizing archaea (marine)** | | | | |
| Mixed culture | 1.114 | 10.0 – 25.0 | 5.9 – 8.7 | ^† (5)^ |
| *Nitrosopumilus maritimus* | 1.120 | 5.0 – 32.0 | 7.3 | [4] |
| **Nitrite-oxidizing bacteria (non-marine)** | | | | |
| Mixed culture | 1.087 | 5.0 – 25.0 | 7.0 – 7.5 | ^† (6)^ |
| *Nitrobacter* species | 1.089 | 5.0 – 25.0 | 7.0 – 7.5 | ^† (7)^ |
| *Nitrospira* species | 1.088 | 5.0 – 25.0 | 7.0 – 7.5 | ^† (8)^ |
| *Ca.* Nitrotoga arctica | 1.109 | 5.0 – 15.0 | 7.0 – 7.5 | ^† (9)^ |
| *Nitrobacter agilis* | 1.104 | 2.5 – 25.0 | 7.8 | [5] |
| *Nitrospira defluvii* | 1.110 | 10.0 – 27.5 | 7.4 | [6] |
| **Nitrite-oxidizing bacteria (marine)** | | | | |
| *Nitrococcus mobilis* | 1.087 | 5.0 – 25.0 | 7.0 – 7.5 | ^† (6)^ |
| *Nitropina* species | 1.087 | 5.0 – 25.0 | 7.0 – 7.5 | ^† (7)^ |
| *Nitrospira* species | 1.088 | 5.0 – 25.0 | 7.0 – 7.5 | ^† (8)^ |
| *Nitrotoga* species | 1.109 | 5.0 – 15.0 | 7.0 – 7.5 | ^† (9)^ |
| ^†^ Estimated  ^(1)^ Average of *Nitrosomonas spp.* (1.116, 1.101, 1.078) and *Nitrosospira spp.* (1.110, 1.146, 1.114, 1.132) [1, 7-9]  ^(2)^ Average of *Nitrosomonas spp.* (1.116, 1.101, 1.078) [7-9]  ^(3)^ Average of *Nitrosospira spp.* (1.110, 1.146, 1.114, 1.132) [1]  ^(4)^ Average of *N aquarius* (1.083), *N viennensis* (1.180) and *Ca.* N. exaquare (1.114) [3, 10, 11]  ^(5)^ Average of *N. maritimus* (1.120), *N. cobalaminigenes* (1.097) and *N. ureiphilus*(1.126) [4]  ^(6)^ Average of *Nitrobacter spp.* (1.104, 1.108, 1.080, 1.064), *Nitrospira spp.* (1.066, 1.110), *Nitrotoga spp.* (1.046, 1.172) and mixed culture of NOB (1.084, 1.036) [5-8, 12-15]  ^(7)^ Average of *Nitrobacter spp.* (1.104, 1.108, 1.080, 1.064) [5, 7, 8, 13]  ^(8)^ Average of *Nitrospira spp.* (1.066, 1.110) [6, 15]  ^(9)^ Average of *Nitrotoga spp.* (1.046, 1.172) [6, 14] | | | | |

**Table S2.** Maximum specific growth rate (µ_max_) values at 20ºC of nitrifying bacteria and archaea

| **Culture** | **µ_max_ (h^-1^)** | **θ^(1)^** | **pH** | **References** |
| --- | --- | --- | --- | --- |
| **Ammonia-oxidizing bacteria (non-marine)** | | | | |
| Mixed culture | 0.033 – 0.042 | 1.114 | 7.5 – 7.8 | [16-19] |
| *Nitrosomonas europaea* | 0.014 – 0.035 | 1.098 | 7.0 – 7.8 | [20-22] |
| *Nitrosomonas oligotropha* | 0.014 – 0.031 | 1.098 | 7.5 | [23, 24] |
| *Nitrosospira sp.* 40K1 | 0.009 | 1.110 | 7.5 | [1] |
| *Nitrosospira sp.* AF | 0.004 | 1.146 | 7.5 | [1] |
| *Nitrosospira sp.* B6 | 0.010 | 1.114 | 7.5 | [1] |
| *Nitrosospira sp.* L115 | 0.010 | 1.132 | 7.5 | [1] |
| **Ammonia-oxidizing bacteria (marine)** | | | | |
| *Nitrosomonas marina* | 0.018 | 1.098 | 7.6 | [25] |
| *Nitrosococcus oceani* | 0.014 | 1.114 | 8.1 | [25] |
| **Ammonia-oxidizing archaea (non-marine)** | | | | |
| Mixed culture | 0.008 - 0.009 | 1.126 | 7.5 |  |
| *Nitrososphaera koreensis* | 0.009 | 1.150 | 6.0 – 8.0 | [2] |
| *Nitrososphaera vienennsis* | 0.002 | 1.180 | 7.6 | [26] |
| *Nitrososphaera gargensis* | 0.002 | 1.126 | 7.6 | [26] |
| **Ammonia-oxidizing archaea (marine)** | | | | |
| Mixed culture | 0.005 – 0.15 | 1.126 | 8.0 – 8.2 | [27, 28] |
| *Nitrosopumilus maritimus* | 0.008 – 0.013 | 1.120 | 7.0 – 7.6 | [29-31] |
| *Nitrosopumilus piranensis* | 0.003 | 1.180 | 7.1 | [32] |
| *Nitrosopumilus adriaticus* | 0.003 | 1.180 | 7.1 | [32] |
| **Complete ammonia-oxidizing bacteria (CMX)** | | | | |
| *Nitrospira inopinata* | 0.002 | 1.076 | 7.6 | [26] |
| **Nitrite-oxidizing bacteria (non-marine)** | | | | |
| *Nitrobacter vulgaris* | 0.027 – 0.038 | 1.089 | 7.4 – 7.6 | [33, 34] |
| *Nitrospira sp.* ND1 | 0.006 | 1.088 | 7.8 – 8.0 | [35] |
| *Nitrospira japonica* | 0.012 | 1.088 | 7.8 – 8.0 | [35] |
| *Ca.* Nitrotoga arctica | 0.0215 | 1.109 | 7.4 – 7.6 | [34] |
| *Nitrobacter agilis* | 0.016 – 0.017 | 1.104 | 7.8 | [5, 36] |
| *Nitrobacter winogradskyi* | 0.005 – 0.034 | 1.089 | 7.3 – 7.8 | [34, 37, 38] |
| *Nitrospira defluvii* | 0.0081 | 1.110 | 7.4 – 7.6 | [34] |
| *Nitrospira* *lenta* | 0.0095 | 1.088 | 7.4 – 7.6 | [34] |
| *Nitrospira moscoviensis* | 0.005 – 0.017 | 1.088 | 7.4 – 7.8 | [34, 39] |
| *Nitrobacter hamburgensis* | 0.005 – 0.088 | 1.089 | 7.3 – 7.6 | [34, 37] |
| **Nitrite-oxidizing bacteria (marine)** | | | | |
| *Nitrococcus mobilis* | 0.033 – 0.036 | 1.087 | 7.6 – 7.8 | [25, 40] |
| *Nitrotoga* sp. AM1 | 0.0194 | 1.109 | 7.8 | [41] |
| *Nitrospira marina* | 0.0153 | 1.088 | 7.8 | [42] |
| *Nitrospina watsonii* | 0.0142 | 1.087 | 8.0 | [43] |
| *Nitrospira sp.* Ecomares 2.1 | 0.004 – 0.012 | 1.088 | 7.5 | [44] |
| ^(1)^ Arrhenius coefficient. Table S1 gathers the valid temperature range to each Arrhenius coefficient and references.  ^(2)^ OMZ: Oxygen Minimum Zone | | | | |

**Table S3.** Specific affinity for oxygen (a^0^_O2_) of nitrifying bacteria and archaea

| **Culture** | **a^0^_O2_ (L/gBio/h)** | **Temperature (ºC)** | **pH** | **References** |
| --- | --- | --- | --- | --- |
| **Ammonia-oxidizing bacteria (non-marine)** | | | | |
| Mixed culture | 90.47 | 25 | 7.5 | [45] |
| *Nitrosomonas europaea* | 121.68 – 2222.22 | 25 | 75 | [27, 46, 47] |
| **Ammonia-oxidizing archaea (non-marine)** | | | | |
| *Nitrosoarchaeum koreensis* | 338.03 | 25 | 7.5 | [2] |
| **Ammonia-oxidizing archaea (marine)** | | | | |
| Mixed culture | 995.02 | 25 | 7.5 | [27] |
| *Nitrosopumilus maritimus* | 1619.43 | 25 | 7.5 | [31] |
| **Nitrite-oxidizing bacteria (non-marine)** | | | | |
| *Nitrobacter hamburgensis* | 23.34 – 63.06 | 25 | 7.5 | [46] |
| *Nitrobacter winogradskyi* | 79.71 – 116.31 | 25 | 7.5 | [47] |
| *Nitrospira sp.* ND1 | 0.05 – 0.21 | 25 | 8.0 | [35] |
| *Nitrospira* *japonica* | 0.05 – 0.21 | 25 | 8.0 | [35] |
| Factors: 1.9mgBio(dry)/mg of protein; 194fgBio(dry)/cell; 3gBio(wet)/gBio(dry); 0.15pg of protein/cell [48, 49] | | | | |

**Table S4.** Specific affinity for ammonia (a^0^_NH3_) of ammonia oxidizing bacteria and archaea

| **Culture** | **a^0^_NH3_ (L/gBio/h)** | **Temperature (ºC)** | **pH** | **References** |
| --- | --- | --- | --- | --- |
| **Ammonia-oxidizing bacteria (non-marine)** | | | | |
| Mixed culture | 29.24 | 20 | 7.7 | [50] |
| *Nitrosomonas europaea* | 3.56 – 38.73 | 25 – 30 | 7.5 – 7.8 | [27, 31, 46, 47] |
| *Nitrosospira sp.* 40K1 | 637.96 | 22 | 7.8 | [1] |
| *Nitrosospira sp.* AF | 1384.02 | 22 | 7.8 | [1] |
| *Nitrosospira sp.* B6 | 628.65 | 22 | 7.8 | [1] |
| *Nitrosospira sp.* L115 | 688.76 | 22 | 7.8 | [1] |
| *Nitrosomonas oligotropha* | 0.0067 – 0.0618 | 27 – 30 | 7.5 – 7.8 | [23] |
| **Ammonia-oxidizing bacteria (marine)** | | | | |
| *Nitrosococcus oceani* | 0.0077 – 0.595 | ? | ? | [31, 51] |
| **Ammonia-oxidizing archaea (non-marine)** | | | | |
| *Nitrosotenuis uzoenensis* | 105.18 – 125.19 | 30 | 7.6 | [26] |
| *Nitrososphaera gargensis* | 773.81 – 1350.35 | 30 | 7.6 | [26] |
| *Nitrososphaera viennensis* | 913.98 – 1169.59 | 30 | 7.6 | [26] |
| *Nitrosoarchaeum koreensis* | 2796.85 | 25 | 7 | [2] |
| **Ammonia-oxidizing archaea (marine)** | | | | |
| *Nitrosopumilus maritimus* | 31120.71 | 30 | 7.5 | [31] |
| **Complete ammonia-oxidizing bacteria (CMX)** | | | | |
| *Nitrospira inopinata* | 2488.04 – 3558.90 | 30 | 7.6 | [26] |
| *Ca.* Nitrospira kreftii | 5864.14 – 7415.09 | 25 | 7.5 | [52] |
| Factors: 1.9mgBio(dry)/mg of protein; 194fgBio(dry)/cell; 3gBio(wet)/gBio(dry); 0.15pg of protein/cell [48, 49] | | | | |

**Table S5.** Specific affinity for nitrite (a^0^_NO2_) of nitrite oxidizing bacteria

| **Culture** | **a^0^_NO2_ (L/gBio/h)** | **Temperature (ºC)** | **pH** | **References** |
| --- | --- | --- | --- | --- |
| **Nitrite-oxidizing bacteria (non-marine)** | | | | |
| *Nitrobacter winogradskyi* | 10.56 – 62.72 | 23 – 30 | 7.3 – 7.6 | [34, 37, 53] |
| *Nitrobacter hamburgensis* | 2.17 – 20.64 | 25 – 28 | 7.3 – 7.5 | [34, 37, 46] |
| *Nitrobacter agilis* | 9.88 – 14.73 | 20 – 30 | 6.5 – 8.5 | [53] |
| *Nitrobacter vulgaris* | 587.18 | 28 | 7.5 | [34] |
| *Nitrospira defluvii* | 935.67 | 28 | 7.5 | [34] |
| *Nitrospira moscoviensis* | 350.88 | 37 | 7.5 | [34] |
| *Nitrospira lenta* | 129.95 | 28 | 7.5 | [34] |
| *Nitrospira japonica* | 543.86 | 25 | 8.0 | [35] |
| *Nitrospira sp.* ND1 | 1315.79 | 25 | 8.0 | [35] |
| *Ca.* Nitrotoga arctica | 78.64 | 17 | 7.5 | [34] |
| **Nitrite-oxidizing bacteria (marine)** | | | | |
| *Nitrosococcus mobilis* | 175.44 | 28 | 7.8 | [54] |
| *Nitrospira sp.* Ecomares 2.1 | 69.53 | 28 | 7.8 | [54] |
| *Nitrotoga sp.* AM1 | 288.85 | 16 | 7.8 | [41] |
| *Nitrospina watsonii* | 345.25 | 28 | 7.8 | [54] |
| Factors: 1.9mgBio(dry)/mg of protein; 194fgBio(dry)/cell; 3gBio(wet)/gBio(dry); 0.15pg of protein/cell [48, 49] | | | | |

**Table S6.** Biomass growth yield (Y_XS_) of ammonia oxidizing bacteria and archaea

| **Culture** | **Y_XS_ (gBio/gNH_3_)** | **Temperature (ºC)** | **pH** | **References** |
| --- | --- | --- | --- | --- |
| **Ammonia-oxidizing bacteria (non-marine)** | | | | |
| Mixed culture | 0.0329 – 0.0107 | 21 – 25 | 7.0 – 8.5 | [17, 55] |
| *Nitrosospira sp.* 40K1 | 0.0326 | 22 | 7.8 | [1] |
| *Nitrosospira sp.* AF | 0.0127 | 22 | 7.8 | [1] |
| *Nitrosospira sp.* B6 | 0.0507 | 22 | 7.8 | [1] |
| *Nitrosospira sp.* L115 | 0.0489 | 22 | 7.8 | [1] |
| *Nitrosomonas europaea* | 0.052 – 0084 | 30 | 8.0 | [22, 26, 56] |
| **Ammonia-oxidizing bacteria (marine)** | | | | |
| *Nitrosococcus oceani* | 0.081, 0.047 | 28 – 30 | 7.5 | [26, 57] |
| **Ammonia-oxidizing archaea (non-marine)** | | | | |
| *Nitrososphaera gargensis* | 0.096 – 0.109 | 30 | 7.6 | [26] |
| *Nitrososphaera viennensis* | 0.096 – 0.103 | 30 | 76 | [26] |
| **Ammonia-oxidizing archaea (marine)** | | | | |
| *Nitrosopumilus maritimus* | 0.076, 0.092, 0.068 | 28 – 30 | 7.5 – 7.8 | [29, 31, 57] |
| *Nitrosopumilus piranensis* | 0.0744 | 32.5 | 7.1 | [32] |
| *Nitrosopumilus adriaticus* | 0.081 | 30 | 7.1 | [32] |
| **Complete ammonia-oxidizing bacteria (CMX)** | | | | |
| *Nitrospira inopinata* | 0.125 – 0.137 | 30 | 7.6 | [26] |
| ^(1)^ Theoretical value. Maximum growth yield.  Factors:1.9mgBio(dry)/mg of protein; 194fgBio(dry)/cell; 3gBio(wet)/gBio(dry); 1.416gCOD/gBio; 0.4gBio(D)/L/OD_600_ [48, 49, 58] | | | | |

**Table S7.** Biomass growth yield (Y_XS_) of nitrite oxidizing bacteria

| **Culture** | **Y_XS_ (gBio/gNO_2_)** | **Temperature  (ºC)** | **pH** | **References** |
| --- | --- | --- | --- | --- |
| **Nitrite-oxidizing bacteria (non-marine)** | | | | |
| *Nitrospira japonica* | 0.043 | 25 | 8.0 | [35] |
| *Nitrobacter agilis* | 0.0126 | 30 | 8.0 | [59] |
| *Nitrobacter winogradskyi* | 0.0103 | 28 | 7.5 | [34] |
| *Nitrospira sp.* ND1 | 0.0258 | 25 | 8.0 | [35] |
| *Nitrospira moscoviensis* | 0.0149 – 0.0264 | 37 | 7.5 – 8.6 | [34, 60] |
| *Nitrobacter hamburgensis* | 0.0133 – 0.0266 | 28 – 30 | 7.5 – 7.8 | [34, 61] |
| *Nitrospira lenta* | 0.0158 | 38 | 7.5 | [34] |
| *Nitrospira defluvii* | 0.0151 | 28 | 7.5 | [34] |
| *Ca.* Nitrotoga arctica | 0.0126 | 17 | 7.5 | [34] |
| *Nitrobacter vulgaris* | 0.0123 | 28 | 7.5 | [34] |
| **Nitrite-oxidizing bacteria (marine)** | | | | |
| *Nitrospira watsonii* | 0.0273 | 28 | ? | [43] |
| *Nitrospira sp.* Ecomares 2.1 | 0.0074 – 0.0372 | 25 | 7.0 – 7.5 | [44] |
| *Nitrospira marina* | 0.005 – 0.0119 | 28 | 7.6 – 8.0 | [62] |
| ^(1)^ Theoretical value. Maximum growth yield  ^(2)^ See Supplementary text – Materials and Methods. OMZ: Oxygen Minimum Zone  Factors: 1.9mgBio(dry)/mg of protein; 194fgBio(dry)/cell; 3gBio(wet)/gBio(dry); 1.416gCOD/gBio [48, 49, 58] | | | | |

**Table S8.** Inventory of the terminal oxidases of ammonia and nitrite oxidisers. The presence of terminal oxidase in each nitrifier groups is indicated with their references

| **Terminal oxidase** | **AOB** | **AOA** | **NOB** | | |
| --- | --- | --- | --- | --- | --- |
|  |  |  | **Nitrobacter** | **Nitrospira** | **Nitrospina** |
| Cytochrome c oxidase aa_3_ | [63-65] | [66, 67] | [68-70] |  |  |
| Putative cytochrome *bd*-like |  |  |  | [71] |  |
| Cytochrome c oxidase cbb_3_ | [64]^(1)^ |  |  |  | [72] |
| ^(1)^ Isolated only in *Nitrosomonas eutropha* and *Nitrosomonas* cluster 7 strain GH22 | | | | |  |

**Table S9.** Intrinsic half-saturation constant for oxygen (K_O2_) of terminal oxidases isolated in nitrifiers

| **Terminal oxidase** | **E^(1)^ (µM)** | **M^(2)^ (µM)** | **L^(3)^ (nM)** | **References** |
| --- | --- | --- | --- | --- |
| Cytochrome c oxidase aa_3_ | 0.97 – 5.00 | ND^(4)^ | 50 – 62 | [73-76] |
| Putative cytochrome *bd*-like | 0.27 – 4.00 | 0.27 – 0.41 | 8 – 24 | [73, 77-85] |
| Cytochrome c oxidase cbb_3_ | 0.08 – 0.98 | 0.011 – 0.151 | 4 – 7 | [73, 76, 86, 87] |
| ^(1)^ Method to determine K_O2_: Oxygen electrode method. Cultures: *P. aerugionsa, B. cereus, E. coli, A. vinelandii. B. japonicum and* mitochondrial c. aa_3_.  ^(2)^ Method to determine K_O2_: Deoxygenation kinetics of oxymyoglobin. Cultures: *P. aerugionsa, A. vinelandii and Rhizobium spp.*  ^(3)^ Method to determine K_O2_: Deoxygenation kinetics of oxyleghemoglobin. Cultures: *B. japonicum, K. pneumoniae, E. coli,*  mitochondrial c. aa_3_, *A. vinelandii, P. aerugionsa* and *Bradyrhizobium spp.*  ^(4)^ ND, not determined | | | | |

**Table S10.** References of apparent substrate affinity (K_m(app)_) for O_2_ of nitrite-oxidizing bacteria (NOB).

| **Genera** | **Growth measurements** | **Activity measurements** | |
| --- | --- | --- | --- |
| *Nitrobacter* | [59, 88-90] | [46, 47, 91] | |
| *Nitrospira* | [92] | [35] | |
| *Nitrospina* | ND^(1)^ | [93] | |
| ^(4)^ ND, not determined | | |  |

**Table S11.** Summary and description of the data collected from literature of considered ammonia oxidizers. References are provided in Supplementary Tables

|  | **C**ᶲ | **Growth rate (µ_max_)** | | | |  | **Ammonia affinity (a^0^_NH3_)** | | | |  | **Oxygen affinity (a^0^_O2_)** | | | |  | **Growth yield (Y_XS_)** | | | |
| --- | --- | --- | --- | --- | --- | --- | --- | --- | --- | --- | --- | --- | --- | --- | --- | --- | --- | --- | --- | --- |
|  |  | **n**^†^ | **SO**^‡^ | **M**^§^ | **I**^¶^ |  | **n** | **SO** | **M** | **I** |  | **n** | **SO** | **M** | **I** |  | **n** | **SO** | **M** | **I** |
| **Ammonia-oxidizing bacteria (non-marine)** | | | | | | | | | | | | | | | | | | | | |
| Mixed culture (Mx AOB-FW) | Mx | 6 | N, W | R, CO, M | S |  | 8 | N, W | R, M, D | S |  | 7 | W | R, M, B | S |  | 7 | N, W | D, B, M, R | S |
| *Nitrosomonas europaea* | P | 7 | N | B, D, P | S |  | 6 | N, W | P, D, B | S |  | 11 | N, W | R, P, M | S |  | 3 | N | D, B, E | S |
| *Nitrosomonas oligotropha* | E, P | 3 | N | P, R | S |  | 2 | N | P, R | S |  | 3 | N, W | R, B | S |  | 0 |  |  |  |
| *Nitrosospira sp. 40K1* | P | 1 | N | D | S |  | 1 | N | D | S |  | 0 |  |  |  |  | 1 | N | D | S |
| *Nitrosospira sp. AF* | P | 1 | N | D | S |  | 1 | N | D | S |  | 0 |  |  |  |  | 1 | N | D | S |
| *Nitrosospira sp. B6* | P | 1 | W | D | S |  | 1 | W | D | S |  | 0 |  |  |  |  | 1 | W | D | S |
| *Nitrosospira sp. L115* | P | 1 | N | D | S |  | 1 | N | D | S |  | 0 |  |  |  |  | 1 | N | D | S |
| **Ammonia-oxidizing bacteria (marine)** | | | | | | | | | | | | | | | | | | | | |
| Mixed culture (Mx AOB-SW) | Mx | 3 | N | M | WM |  | 3 | N | M | WM |  | 0 |  |  |  |  | 0 |  |  |  |
| *Nitrosomonas marina* | P | 1 | N | B | S |  | 0 |  |  |  |  | 0 |  |  |  |  | 0 |  |  |  |
| *Nitrosococcus oceani* | P | 1 | N | B | S |  | 4 | N | B | S |  | 0 |  |  |  |  | 2 | N | D, B | S |
| **Ammonia-oxidizing archaea (non-marine)** | | | | | | | | | | | | | | | | | | | | |
| Mixed culture (Mx AOA-FW) | Mx | 2 | N | P | S |  | 2 | N | R | S |  | 1 | N | R | S |  | 0 |  |  |  |
| *Nitrososphaera koreensis* | E | 1 | N | R, P | S |  | 1 | N | R | S |  | 1 | N | R | S |  | 0 |  |  |  |
| *Nitrososphaera vienennsis* | P | 1 | N | P | S |  | 3 | N | R | S |  | 1 | N | R | S |  | 4 | N | PC | S |
| *Nitrososphaera gargensis* | P | 1 | N | P | S |  | 2 | N | R | S |  | 0 |  |  |  |  | 4 | N | PC | S |
| *Nitrosotenuis uzoenensis* | E | 0 |  |  |  |  | 2 | N | R | S |  | 0 |  |  |  |  | 0 |  |  |  |
| **Ammonia-oxidizing archaea (marine)** | | | | | | | | | | | | | | | | | | | | |
| Mixed culture (Mx AOA‑SW) | Mx | 4 | N | R, P | NM |  | 1 | N | R | NM |  | 1 | N | R | NM |  | 0 |  |  |  |
| Mixed culture from OMZ^(1)^ | Mx | 0 |  |  |  |  | 0 |  |  |  |  | 2 | N | R | NM |  | 0 |  |  |  |
| *Nitrosopumilus maritimus* | P | 3 | N | R | S |  | 2 | N | R, B | S |  | 2 | N | R | S |  | 3 | N | D, E | S |
| *Nitrosopumilus piranensis* | E | 1 | N | E | S |  | 0 |  |  |  |  | 0 |  |  |  |  | 1 | N | E | S |
| *Nitrosopumilus adiactus* | E | 1 | N | E | S |  | 0 |  |  |  |  | 0 |  |  |  |  | 1 | N | E | S |
| **Complete ammonia-oxidizing bacteria (CMX)** | | | | | | | | | | | | | | | | | | | | |
| *Nitrospira inopinata* | *P* | *1* | *N* | *P* | *S* |  | *1* | *N* | *R* | *S* |  | *0* |  |  |  |  | *4* | *N* | *PC* | *S* |
| *Ca.* Nitrospira kreftii | E | *0* |  |  |  |  | *1* | *N* | *R* | *S* |  | *0* |  |  |  |  | *0* |  |  |  |
| ᶲ C = Culture type: Mx – Mixed culture; P – Pure culture; E – Enriched culture  ^†^ n = Number of parameters  ^‡^ SO **=** Sample origin: N – Natural environment; W – Wastewater treatment plant  ^§^ M = Experimental method: R – Respirometry; D – Cell density/Cell count; CO – Maximum CO_2_ uptake rate; M – Model; B – Bibliographia; P – Product/substrate rates or log plot; PC – Protein content; E – Estimated (Y_XS_ = ΔX/ΔS)  ^¶^ I = Medium: S – Synthetic medium; NM – Natural medium; WM – Real wastewater  ^(1)^ OMZ: Oxygen Minimum Zone | | | | | | | | | | | | | | | | | | | | |

**Table S12.** Summary and description of the data collected from literature of considered nitrite oxidizers. References are provided in Supplementary Tables

|  | **C**ᶲ | **Growth rate (µ_max_)** | | | |  | **Nitrite affinity (a^0^_NO2_)** | | | |  | **Oxygen affinity (a^0^_O2_)** | | | |  | **Growth yield (Y_XS_)** | | | |
| --- | --- | --- | --- | --- | --- | --- | --- | --- | --- | --- | --- | --- | --- | --- | --- | --- | --- | --- | --- | --- |
|  |  | **n**^†^ | **SO**^‡^ | **M**^§^ | **I**^¶^ |  | **n** | **SO** | **M** | **I** |  | **n** | **SO** | **M** | **I** |  | **n** | **SO** | **M** | **I** |
| **Nitrite-oxidizing bacteria (non-marine)** | | | | | | | | | | | | | | | | | | | | |
| Mixed culture (Mx NOB-FW) | Mx | 5 | N, W | M, D | S |  | 5 | N, W | R, D, M | S |  | 13 | N, W | M, B, R | S |  | 6 | N, W | M, D, B | S |
| *Nitrobacter vulgaris* | P | 2 | W | D | S |  | 1 | W | R | S |  | 0 |  |  |  |  | 1 | W | PC | S |
| *Nitrospira sp.* ND1 | P | 1 | W | D | S |  | 1 | W | P | S |  | 2 | W | R | S |  | 1 | W | E | S |
| *Nitrospira japonica* | P | 1 | W | D | S |  | 1 | W | P | S |  | 2 | W | R | S |  | 1 | W | E | S |
| *Ca.* Nitrotoga arctica | P | 1 | N | D | S |  | 0 |  |  |  |  | 0 |  |  |  |  | 1 | N | PC | S |
| *Nitrobacter agilis* | P | 2 | W | M | S |  | 18 | N, W | R | S |  | 2 | N, W | B, M | S |  | 1 | N | M | S |
| *Nitrobacter winogradskyi* | P | 6 | N | D, M | S |  | 10 | N, W | M, R | S |  | 2 | N, W | R, P | S |  | 2 | N, W | PC, M | S |
| *Nitrospira defluvii* | P | 1 | W | D | S |  | 1 | W | R | S |  | 0 |  |  |  |  | 1 | W | PC | S |
| *Nitrospira lenta*^(2)^ | P | 1 | W | D | S |  | 0 |  |  |  |  | 0 |  |  |  |  | 1 | W | PC | S |
| *Nitrospira moscoviensis* | P | 2 | N, W | D, M | S |  | 1 | N, W | M, R | S |  | 0 |  |  |  |  | 2 | N | PC, E | S |
| *Nitrobacter hamburgensis* | P | 5 | N | D | S |  | 5 | N | R | S |  | 2 | N | R | S |  | 2 | N | PC, E | S |
| **Nitrite-oxidizing bacteria (marine)** | | | | | | | | | | | | | | | | | | | | |
| Mixed culture (Mx NOB-SW) | Mx | 0 |  |  |  |  | 0 |  |  |  |  | 1 | W | R | S |  | 0 |  |  |  |
| *Nitrococcus mobilis* | P | 2 | N | D | S |  | 2 | N | R | S |  | 0 |  |  |  |  | 0 |  |  |  |
| *Nitrotoga sp.* AM1 | E | 1 | N | D | S |  | 0 |  |  |  |  | 0 |  |  |  |  | 0 |  |  |  |
| *Nitrospira marina* | P | 1 | N | D | S |  | 0 |  |  |  |  | 0 |  |  |  |  | 1 | N | E | S |
| *Nitrospina gracilis* | p | 1 | N | D | S |  | 0 |  |  |  |  | 0 |  |  |  |  | 0 |  |  |  |
| *Nitrospina watsonii* | E | 1 | N | D | S |  | 2 | N | R | S |  | 0 |  |  |  |  | 1 | N | E | S |
| *Nitrospinae* phyl. from OMZ^(1)^ | Mx | 2 | N | D | NM |  | 2 | N | M | NM |  | 2 | N | R | NM |  | 0 |  |  |  |
| *Nitrospira sp.* Ecomares 2.1 | P | 2 | W | D | NM |  | 2 | W | R | S |  | 0 |  |  |  |  | 2 | W | D, E | NM |
| ᶲ C = Culture type: Mx – Mixed culture; P – Pure culture; E – Enriched culture  ^†^ n = Number of parameters  ^‡^ SO **=** Sample origin: N – Natural environment; W – Wastewater treatment plant  ^§^ M = Experimental method: R – Respirometry; D – Cell density/Cell count; CO – Maximum CO_2_ uptake rate; M – Model; B – Bibliographia; P – Product/substrate rates or log plot; PC – Protein content; E – Estimated (Y_XS_ = ΔX/ΔS)  ^¶^ I = Medium: S – Synthetic medium; NM – Natural medium; WM – Real wastewater  ^(1)^ OMZ: Oxygen Minimum Zone | | | | | | | | | | | | | | | | | | | | |

**Supplementary Figures**

**A**


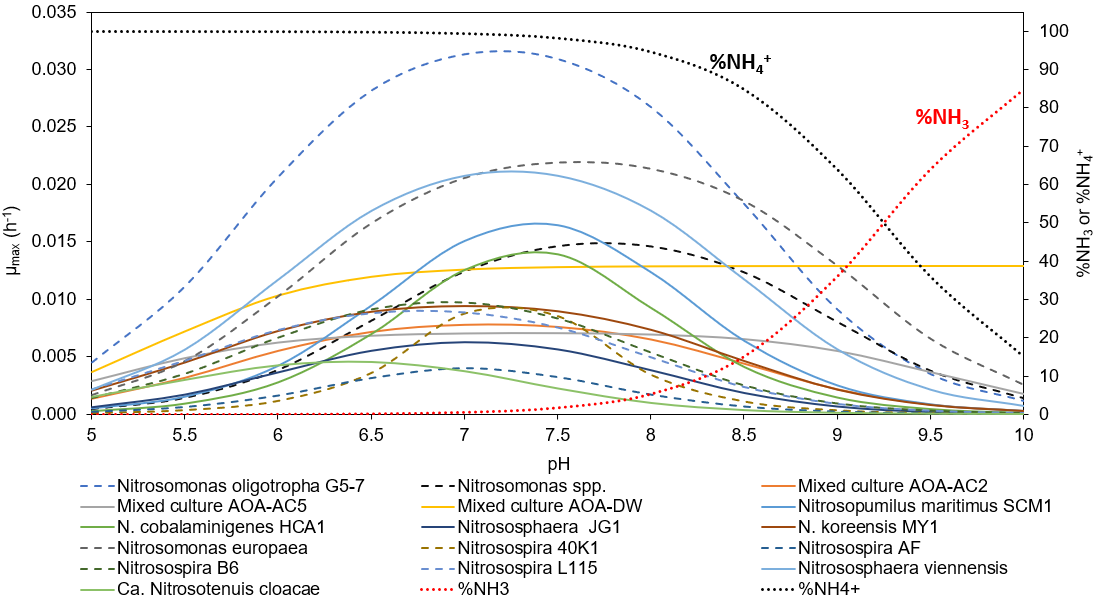


**B**


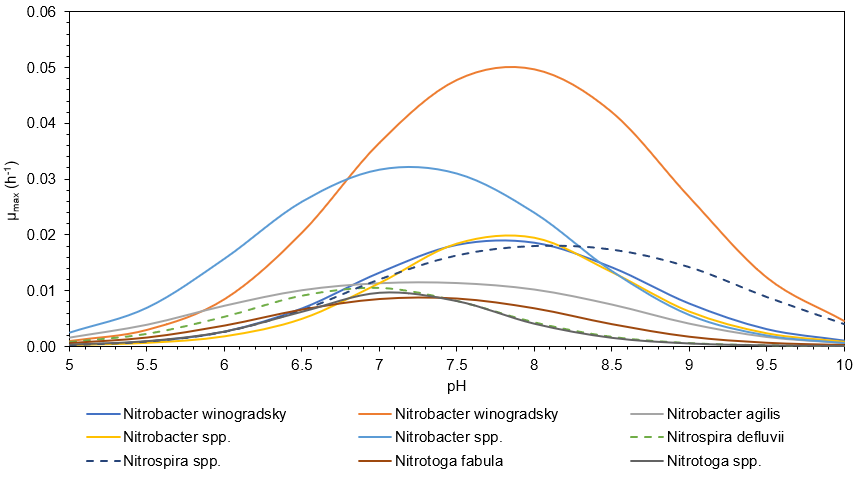


**Figure S1. A)** Maximum specific growth rate of (µ^max^) of AOB and AOA as a function of the pH. Dashed lines represent AOA and solid lines represent AOB. Doted red line represents the percentage of free ammonia (NH_3_) and doted black line represents the percentage of ammonium (NH_4_^+^) in function of pH. References: [1-4, 24, 94-97]. **B)** Maximum specific growth rate of (µ^max^) of NOB as function of the pH. Dashed lines represent species belonging of genus *Nitrospira* and solid lines represent species belonging of genus *Nitrobacter*. References: [6, 14, 15, 22, 36, 91, 98, 99]

| **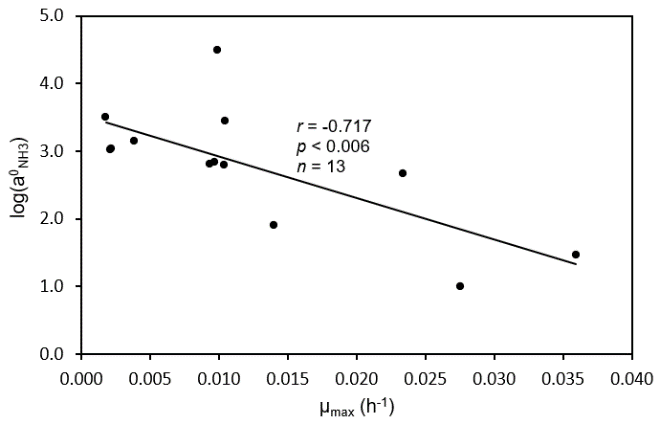**  **A** | **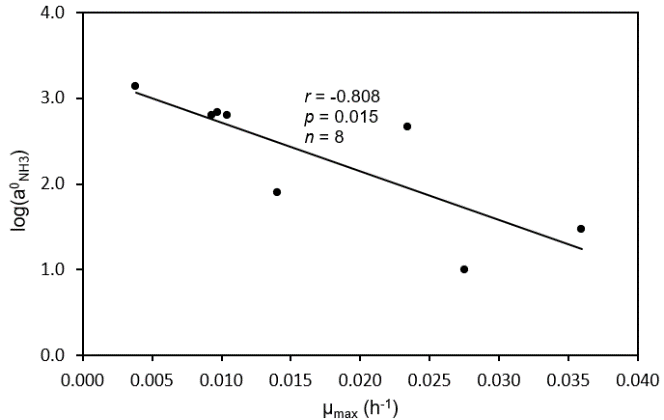**  **B** | **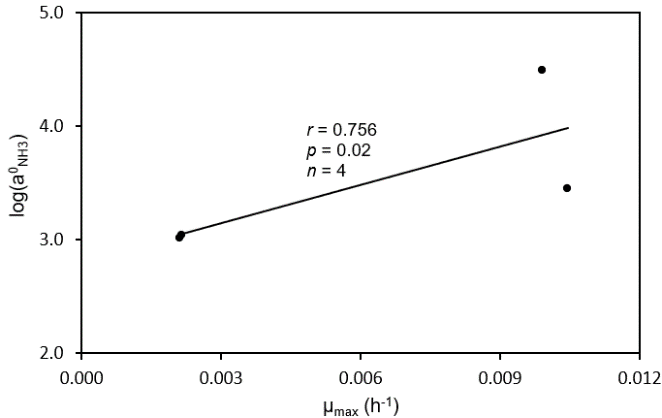**  **C** |
| --- | --- | --- |
| **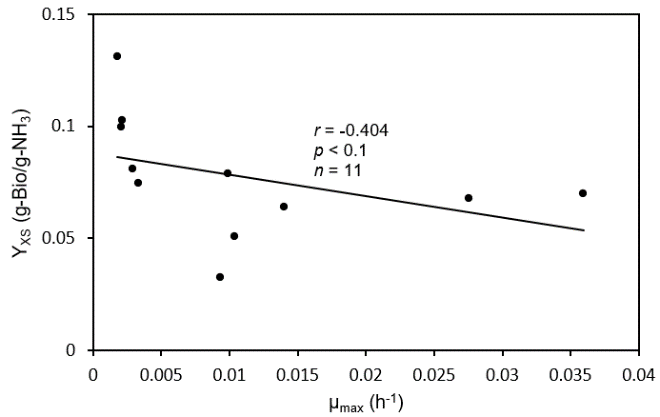**  **D** | **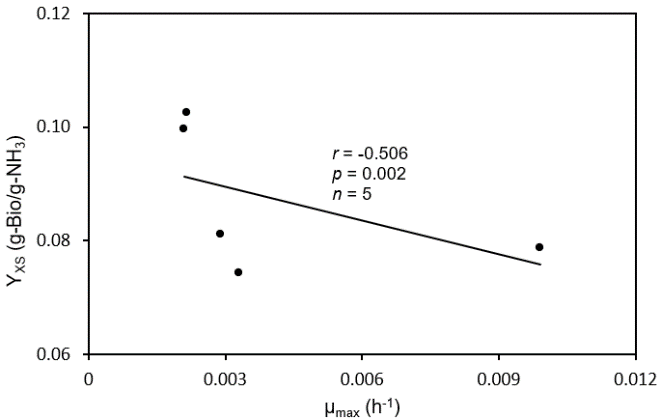**  **E** | **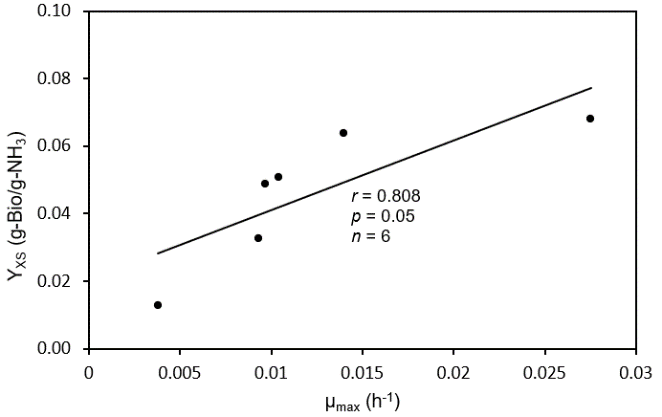**  **F** |
| **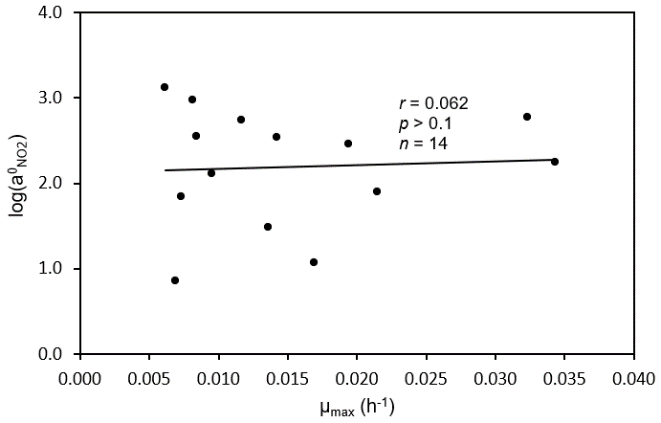**  **G** | **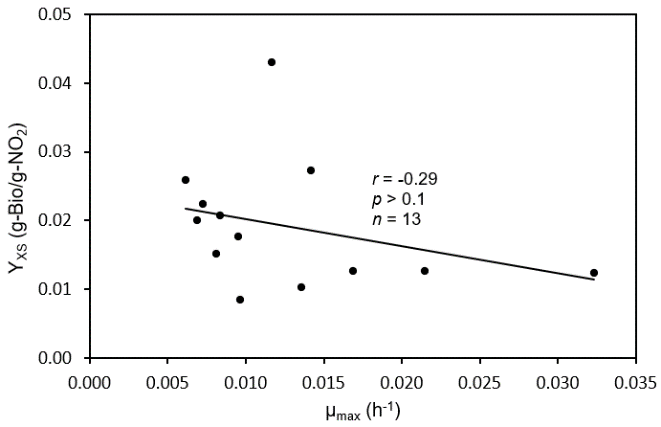**  **H** | **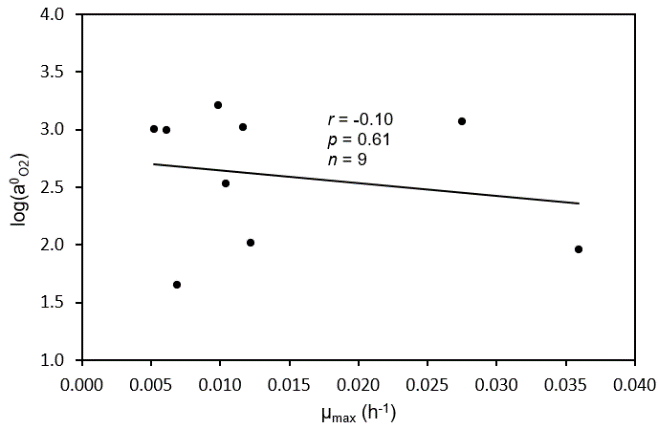**  **I** |

**Figure S2.** Regression fits to different kinetic datasets of nitrifiers and their corresponding Pearson correlation coefficient (*r*) with significance (*p* value) and sample size (*n*). **A)** µ_max_ versus a^0^_NH3_ of AOB, AOA and CMX **B)** µ_max_ versus a^0^_NH3_ of AOB only **C)** µ_max_ versus a^0^_NH3_ of AOA only **D)** µ_max_ versus Y_XS_ of AOB, AOA and CMX (excluding acidophilic AOB) **E)** µ_max_ versus Y_XS_ of AOA only **F)** µ_max_ versus Y_XS_ of AOB only **G)** µ_max_ versus a^0^_NO2_ of NOB **H)** µ_max_ versus Y_XS_ of NOB **I)** µ_max_ versus a^0^_NO2_ of AOB, AOA and NOB


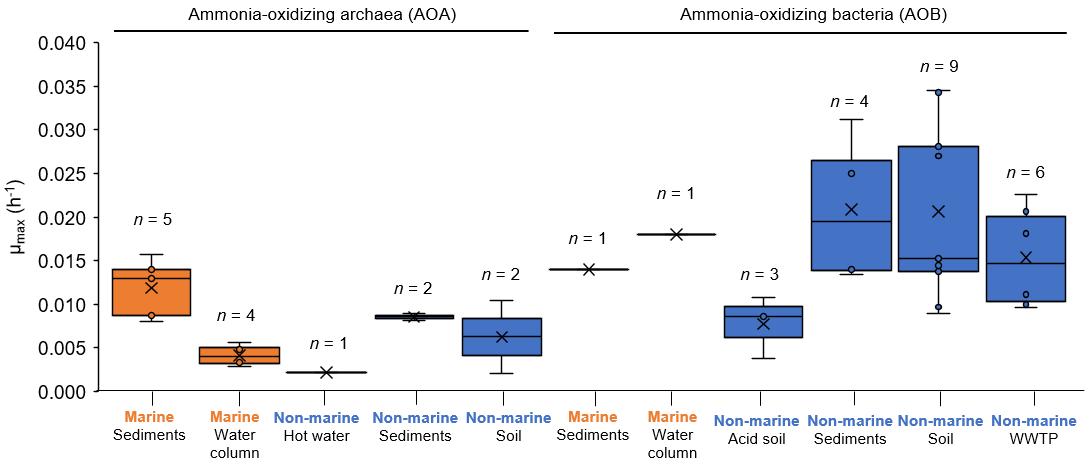


| ***P <0.05 (*)*** | **AOA-Sedim** | **AOA-WatCol** | **AOA-HotWat** | **AOA-Sedim** | **AOA-Soil** | **AOB–Sedim** | **AOB-WatCol** | **AOB-AcidSoil** | **AOB-Sedim** | **AOB-Soil** | **AOB-WWTP** |
| --- | --- | --- | --- | --- | --- | --- | --- | --- | --- | --- | --- |
| **AOA-Sedim** | – | – | – | – | – | – | – | – | – | – | – |
| **AOA-WatCol** | <0.0007* | – | – | – | – | – | – | – | – | – | – |
| **AOA-HotWat** | <0.0001* | >0.05 | – | – | – | – | – | – | – | – | – |
| **AOA-Sedim** | 0.07 | 0.049* | 0.007* | – | – | – | – | – | – | – | – |
| **AOA-Soil** | 0.016* | >0.05 | 0.072 | 0.43 | – | – | – | – | – | – | – |
| **AOB-Sedim** | 0.256 | <0.0001* | <0.0001* | 0.011* | <0.0007* | – | – | – | – | – | – |
| **AOB-WatCol** | 0.007* | <0.0001* | <0.0001* | <0.0001* | <0.0001* | 0.04* | **–** | – | – | – | – |
| **AOB-AcidSoil** | 0.069 | 0.136 | 0.017* | >0.05 | >0.05 | <0.006* | <0.0001* | **–** | **–** | **–** | – |
| **AOB-Sedim** | <0.0001* | <0.0001* | <0.0001* | <0.0001* | <0.0001* | 0.003* | 0.264 | <0.0001* | – | – | – |
| **AOB-Soil** | <0.0001* | <0.0001* | <0.0001* | <0.0001* | <0.0001* | <0.003* | >0.05 | <0.0001* | >0.05 | – | – |
| **AOB-WWTP** | 0.148 | <0.0001* | <0.0001* | 0.002 | <0.0001* | >0.05 | >0.05 | <0.0008* | 0.018* | 0.05 | – |

**Figure S3.** AOB and AOA maximum growth rates (µ_max_) from distinct environments. *P*-values (One-way ANOVA) between environments on bottom table. Number of bibliographic data is indicated above each boxplot. Boxplots depict the 75-100% quantile range, with the centre line depicting the median (50% quantile) and crosses depicting the average value. Data of each environment are shown as points. Table legend: Sedim – Sediments; WatCol – Water column; HotWat – Hot water; AcidSoil – Acidic soil; WWTP ­– Wastewater Treatment Plant.


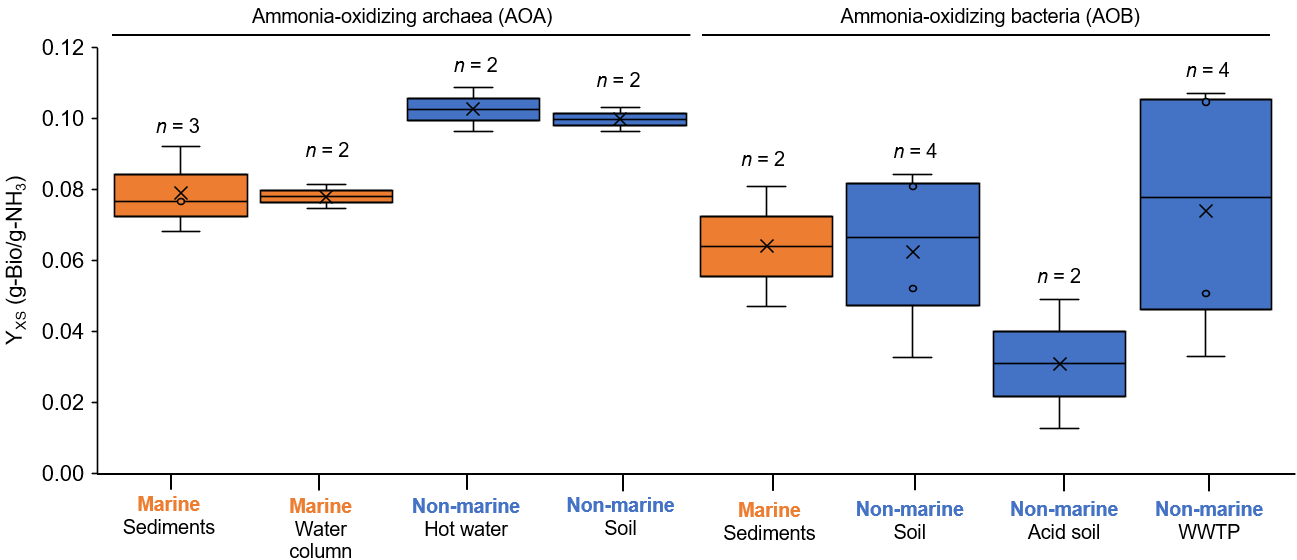


| ***P <0.05 (*)*** | **AOA-Sediments** | **AOA-Water column** | **AOA-Hot water** | **AOA-Soil** | **AOB-Sediments** | **AOB-Soil** | **AOB-Acidic soil** | **AOB-WWTP** |
| --- | --- | --- | --- | --- | --- | --- | --- | --- |
| **AOA-Sediments** | – | – | – | – | – | – | – | – |
| **AOA-Water column** | >0.05 | – | – | – | – | – | – | – |
| **AOA-Hot water** | <0.04* | <0.05* | – | – | – | – | – | – |
| **AOA-Soil** | 0.031* | 0.04* | >0.05 | – | – | – | – | – |
| **AOB-Sediments** | 0.38 | 0.30 | <0.003* | 0.004* | – | – | – | – |
| **AOB-Soil** | 0.69 | 0.06 | <0.0001* | <0.0002* | >0.05 | – | – | – |
| **AOB-Acidic soil** | 0.01* | <0.009* | <0.0002* | <0.0003* | <0.04* | <0.02* | – | – |
| **AOB-WWTP** | 0.85 | >0.05 | 0.03* | 0.04* | >0.05 | >0.05 | <0.02* | – |

**Figure S4.** AOB and AOA growth yields (Y_XS_) from distinct environments. *P*-values (One-way ANOVA) between environments on bottom table. Number of bibliographic data is indicated above each boxplot. Boxplots depict the 75-100% quantile range, with the centre line depicting the median (50% quantile) and crosses depicting the average value. Data of each environment are shown as points. Table legend: WWTP ­– Wastewater Treatment Plant.


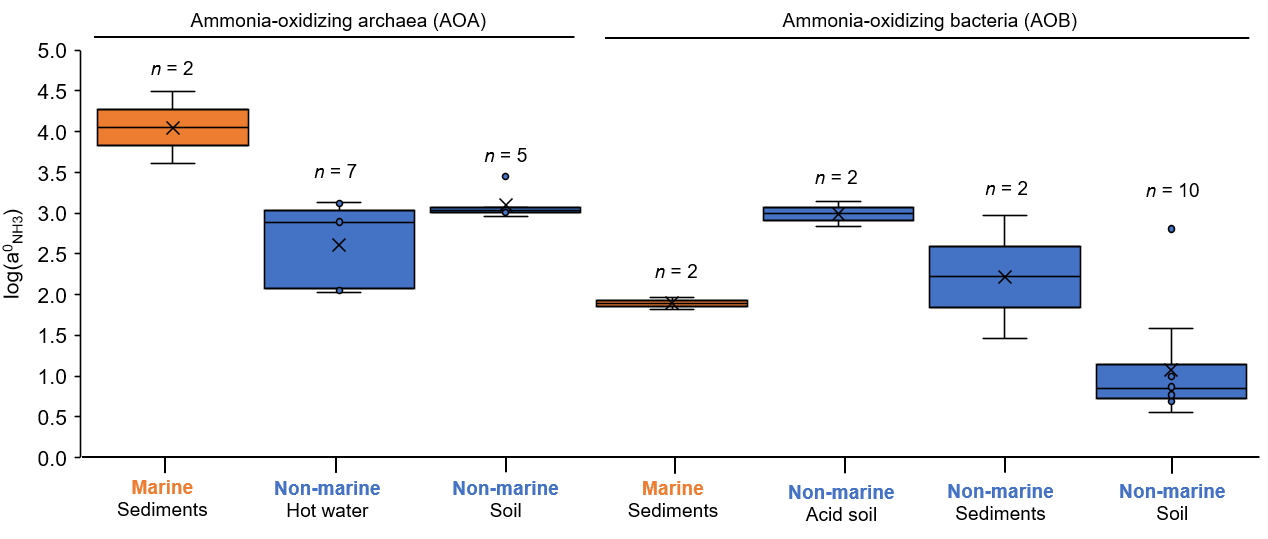


| ***P <0.05 (*)*** | **AOA-Sediments** | **AOA-Hot water** | **AOA-Soil** | **AOB-Sediments** | **AOB-Acidic soil** | **AOB-Sediments** | **AOB-Soil** |
| --- | --- | --- | --- | --- | --- | --- | --- |
| **AOA-Sediments** | – | – | – | – | – | – | – |
| **AOA-Hot water** | <0.0001* | – | – | – | – | – | – |
| **AOA-Soil** | <0.0001* | <0.006* | – | – | – | – | – |
| **AOB-Sediments** | <0.0001* | <0.0001* | <0.0001* | – | – | – | – |
| **AOB-Acidic soil** | <0.0001* | 0.16 | 0.465 | <0.0001* | – | – | – |
| **AOB-Sediments** | <0.0001* | <0.013* | <0.0001* | 0.058 | <0.0001* | – | – |
| **AOB-Soil** | <0.0001* | <0.0001* | <0.0001* | <0.0001* | <0.0001* | <0.0001* | – |

**Figure S5.** AOB and AOA specific affinity for ammonia (a^0^_NH3_) from distinct environments. *P*-values (One-way ANOVA) between environments on bottom table. Number of bibliographic data is indicated above each boxplot. Boxplots depict the 75-100% quantile range, with the centre line depicting the median (50% quantile) and crosses depicting the average value. Data of each environment are shown as points. Values of a^0^_NH3_ were log_10_ transformed before statistical analysis due to the order of magnitude difference in the determined values.


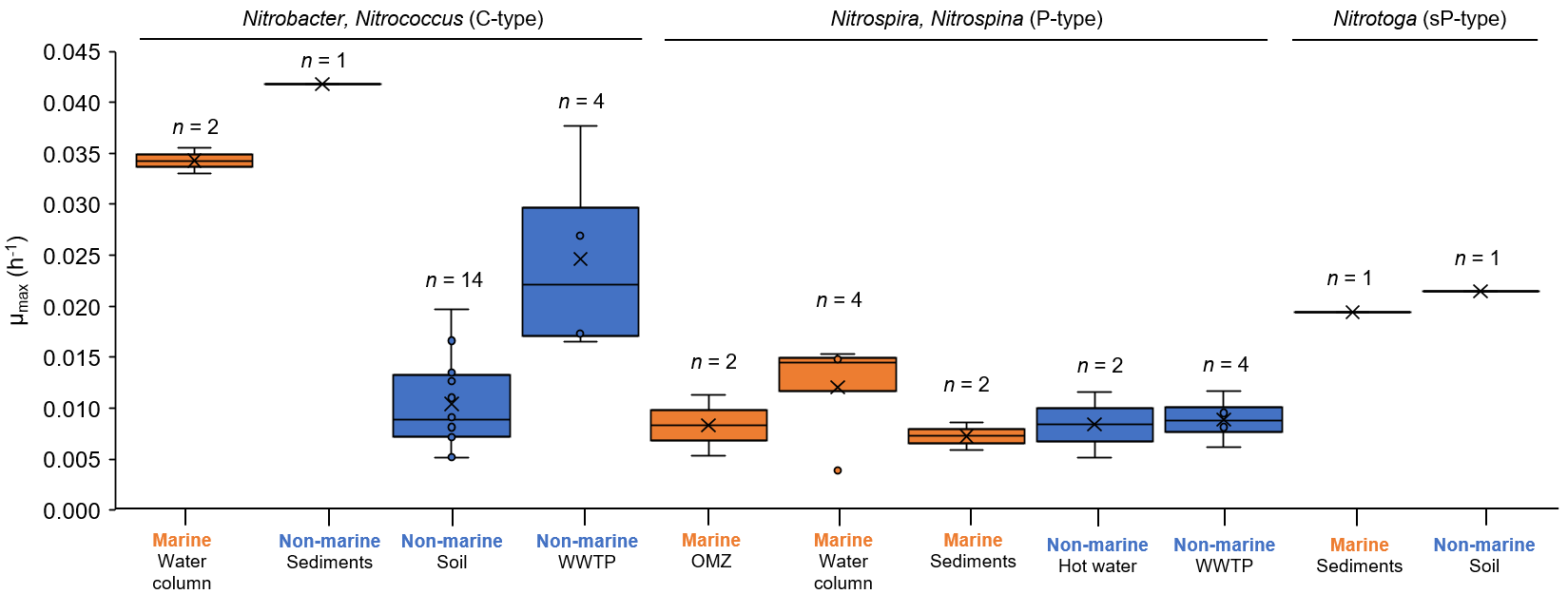


| ***P <0.05 (*)*** | **C–Water col.** | **C–Sediments** | **C–Soil** | **C–WWTP** | **P–OMZ** | **P–Water col.** | **P–Sediments** | **P–Hot water** | **P–WWTP** | **sP–Sediments** | **sP–Soil** |
| --- | --- | --- | --- | --- | --- | --- | --- | --- | --- | --- | --- |
| **C–Water col.** | – | – | – | – | – | – | – | – | – | – | – |
| **C–Sediments** | <0.0001* | – | – | – | – | – | – | – | – | – | – |
| **C–Soil** | <0.0001* | <0.0001* | – | – | – | – | – | – | – | – | – |
| **C–WWTP** | <0.0001* | <0.0001* | <0.0001* | – | – | – | – | – | – | – | – |
| **P–OMZ** | <0.0001* | <0.0001* | 0.073 | <0.0001* | – | – | – | – | – | – | – |
| **P–Water col.** | <0.0001* | <0.0001* | 0.059 | <0.0001* | 0.0002* | – | – | – | – | – | – |
| **P–Sediments** | <0.0001* | <0.0001* | 0.003* | <0.0001* | >0.05 | <0.0001* | – | – | – | – | – |
| **P–Hot water** | <0.0001* | <0.0001* | 0.059 | <0.0001* | >0.05 | 0.0002* | >0.05 | – | – | – | – |
| **P–WWTP** | <0.0001* | <0.0001* | 0.069 | <0.0001* | >0.05 | 0.0008* | 0.249 | >0.05 | – | – | – |
| **sP–Sediments** | <0.0001* | <0.0001* | <0.0001* | <0.0001* | <0.0001* | <0.0001* | <0.0001* | <0.0001* | <0.0001* | – | – |
| **sP–Soil** | <0.0001* | <0.0001* | <0.0001* | <0.0004* | <0.0001* | <0.0001* | <0.0001* | <0.0001* | <0.0001* | 0.017* | – |

**Figure S6.** NOB maximum growth rates (µ_max_) from distinct environments. *P*-values (One-way ANOVA) between environments on bottom table. Number of bibliographic data is indicated above each boxplot. Boxplots depict the 75-100% quantile range, with the centre line depicting the median (50% quantile) and crosses depicting the average value. Data of each environment are shown as points. Table legend: OMZ – Oxygen Minimum Zone; Water col. – Water column; WWTP ­– Wastewater Treatment Plant.


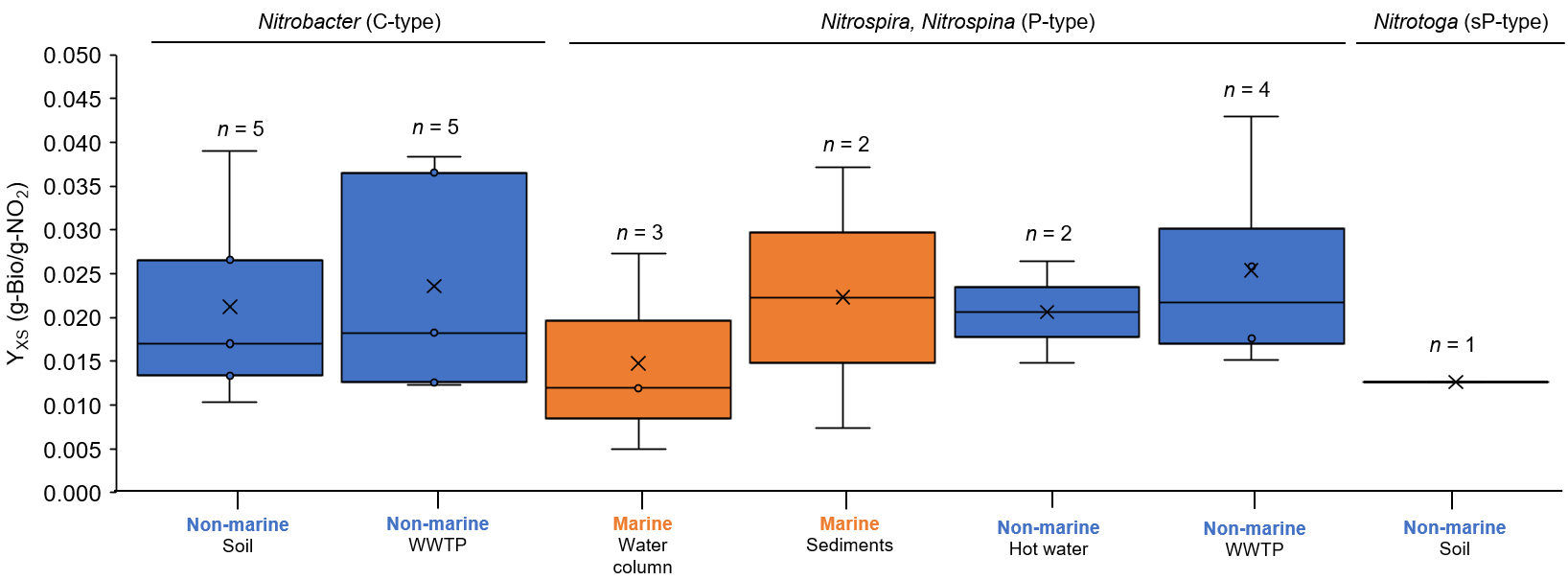


| ***P <0.05 (*)*** | **C-Soil** | **C-WWTP** | **P-Water column** | **P-Sediments** | **P-Hot water** | **P-WWTP** | **sP-Soil** |
| --- | --- | --- | --- | --- | --- | --- | --- |
| **C-Soil** | – | – | – | – | – | – | – |
| **C-WWTP** | >0.1 | – | – | – | – | – | – |
| **P-Water column** | >0.1 | >0.1 | – | – | – | – | – |
| **P-Sediments** | >0.1 | >0.1 | >0.1 | – | – | – | – |
| **P-Hot water** | >0.1 | >0.1 | >0.1 | >0.1 | – | – | – |
| **P-WWTP** | >0.1 | >0.1 | >0.1 | >0.1 | >0.1 | – | – |
| **sP-Soil** | >0.1 | >0.1 | >0.1 | >0.1 | >0.1 | >0.1 | – |

**Figure S7.** NOB growth yields (Y_XS_) from distinct environments. *P*-values (One-way ANOVA) between environments on bottom table. Number of bibliographic data is indicated above each boxplot. Boxplots depict the 75-100% quantile range, with the centre line depicting the median (50% quantile) and crosses depicting the average value. Data of each environment are shown as points. Table legend: WWTP ­– Wastewater Treatment Plant.


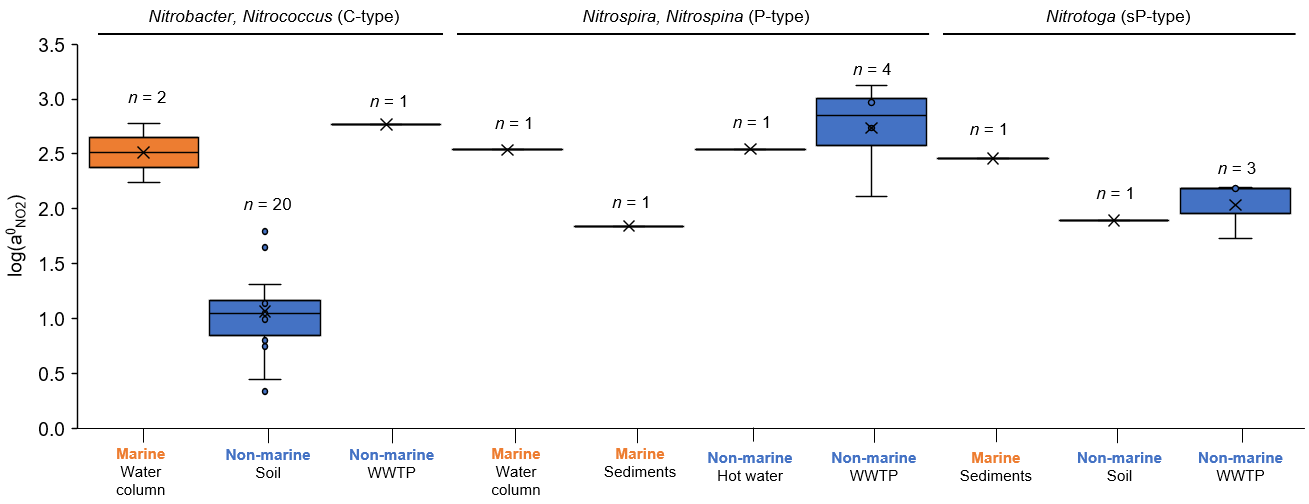


| ***P <0.05 (*)*** | **C-Water column** | **C-Soil** | **C-WWTP** | **P-Water col.** | **P-Sediments** | **P-Hot water** | **P-WWTP** | **sP-Sediments** | **sP-Soil** | **sP-WWTP** |
| --- | --- | --- | --- | --- | --- | --- | --- | --- | --- | --- |
| **C-Water column** | – | – | – | – | – | – | – | – | – | – |
| **C-Soil** | <0.0001* | – | – | – | – | – | – | – | – | – |
| **C-WWTP** | <0.0001* | <0.0001* | – | – | – | – | – | – | – | – |
| **P-Water column** | >0.05 | <0.0001* | <0.0001* | – | – | – | – | – | – | – |
| **P-Sediments** | <0.0001* | <0.0001* | <0.0001* | <0.0001* | – | – | – | – | – | – |
| **P-Hot water** | 0.73 | <0.0001* | <0.0001* | 0.87 | <0.0001* | – | – | – | – | – |
| **P-WWTP** | <0.0001* | <0.0001* | 0.43 | <0.0001* | <0.0001* | <0.0001* | – | – | – | – |
| **sP-Sediments** | 0.23 | <0.0001* | <0.0001* | 0.17 | <0.0001* | >0.20 | <0.0001* | – | – | – |
| **sP-Soil** | <0.0001* | <0.0001* | <0.0001* | <0.0001* | >0.2 | <0.0001* | <0.0001* | <0.0001* | – | – |
| **sP-WWTP** | <0.0001* | <0.0001* | <0.0001* | <0.0001* | <0.0001* | <0.0001* | <0.0001* | <0.0001* | <0.002* | – |

**Figure S8.** NOB specific affinity for nitrite (a^0^_NO2_) from distinct environments. *P*-values (One-way ANOVA) between environments on bottom table. Number of bibliographic data is indicated above each boxplot. Boxplots depict the 75-100% quantile range, with the centre line depicting the median (50% quantile) and crosses depicting the average value. Data of each environment are shown as points. Values of a^0^_NO2_ were log_10_ transformed before statistical analysis due to the order of magnitude difference in the determined values. Table legend: WWTP ­– Wastewater Treatment Plant.


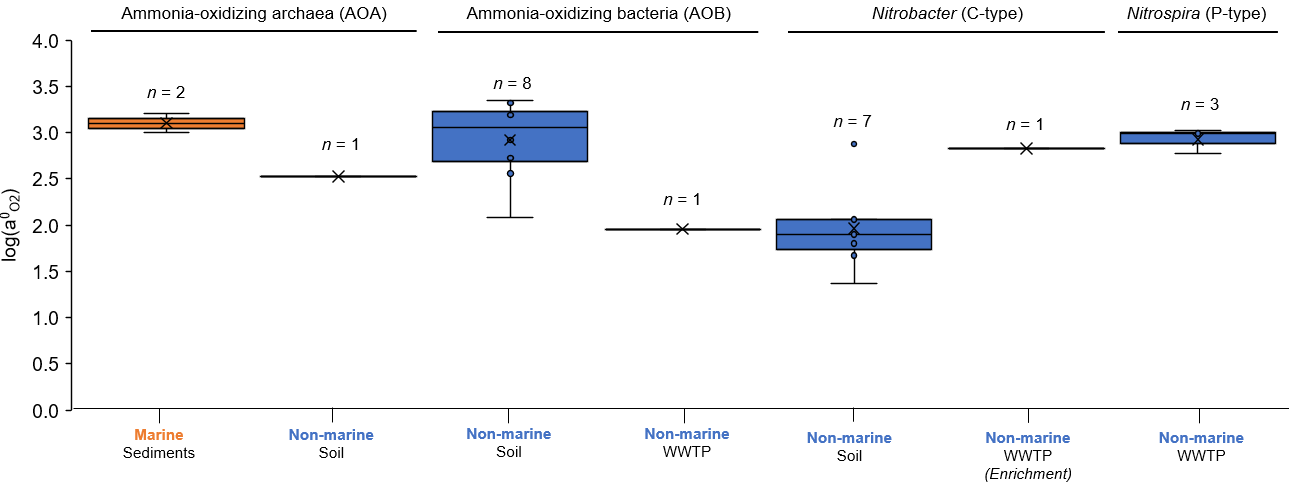


| ***P <0.05 (*)*** | **AOA-Sediments** | **AOA-Soil** | **AOB-Soil** | **AOB-WWTP** | **C-NOB-Soil** | **C-NOB-WWTP^(Enr)^** | **P-NOB-WWTP** |
| --- | --- | --- | --- | --- | --- | --- | --- |
| **AOA-Sediments** | – | – | – | – | – | – | – |
| **AOA-Soil** | <0.0001* | – | – | – | – | – | – |
| **AOB-Soil** | >0.05 | <0.005* | – | – | – | – | – |
| **AOB-WWTP** | <0.0001* | <0.0001* | <0.0001* | – | – | – | – |
| **C-NOB-Soil** | <0.0001* | <0.0001* | <0.0001* | 0.95 | – | – | – |
| **C-NOB-WWTP^(Enr)^** | 0.1 | <0.02* | 0.43 | <0.0001* | <0.0001* | – | – |
| **P-NOB-WWTP** | >0.05 | <0.008* | >0.05 | <0.0001* | <0.0001* | 0.67 | – |

**Figure S9.** AOB, AOA and NOB specific affinity for oxygen (a^0^_O2_) from distinct environments. *P*-values (One-way ANOVA) between environments on bottom table. Number of bibliographic data is indicated above each boxplot. Boxplots depict the 75-100% quantile range, with the centre line depicting the median (50% quantile) and crosses depicting the average value. Data of each environment are shown as points. Values of a^0^_NO2_ were log_10_ transformed before statistical analysis due to the order of magnitude difference in the determined values. Table legend: WWTP ­– Wastewater Treatment Plant.

**Supplementary References**

1. Jiang, Q., *Comparison of Nitrosospira strains isolated from terrestrial environments.* FEMS Microbiology Ecology, 1999. **30**(2): p. 171-186.

2. Jung, M.-y., et al., *Enrichment and Characterization of an Autotrophic Ammonia-Oxidizing Archaeon of Mesophilic Crenarchaeal Group I . 1a from an Agricultural Soil ᰔ †.* 2011. **77**(24): p. 8635-8647.

3. Tourna, M., et al., *Nitrososphaera viennensis, an ammonia oxidizing archaeon from soil.* Proceedings of the National Academy of Sciences of the United States of America, 2011. **108**(20): p. 8420-8425.

4. Qin, W., et al., *Marine ammonia-oxidizing archaeal isolates display obligate mixotrophy and wide ecotypic variation.* 2014. **111**(34).

5. Leenen, E.J.T.M., et al., *Reduced temperature sensitivity of immobilized Nitrobacter agilis cells caused by diffusion limitation.* 1997. **0229**(96): p. 573-580.

6. Wegen, S., B. Nowka, and E. Spieck, *Low temperature andneutral pH define “Candidatus Nitrotoga sp." as a competitive nitrete oxidizier in coculture with Nitrospira defluvii.* 2019. **85**(9): p. 1-10.

7. Helder, W., *Estuarine nitrite maixma and nitrifying bacteria.* 1983. **17**(1): p. 1-18.

8. Knowles, B.Y.G., et al., *Determination of Kinetic Constants for Nitrifying Bacteria in Mixed Culture , with the Aid of an Electronic Computer.* 1965(1965): p. 268-278.

9. Buswell, A.M., et al., *Laboratory Studies on the Kinetics of the Growth of Nitroso- monas with Relation to the Nitrification Phase of the B . O . D . Test.* 1953(3): p. 21-25.

10. Sauder, L.A., et al., *"Candidatus Nitrosotenuis aquarius", an ammonia-oxidizing archaeon from a freshwater aquarium biofilter.* 2018. **84**(19): p. 1-17.

11. Sauder, L.A., et al., *Cultivation and characterization of Candidatus Nitrosocosmicus exaquare , an ammonia-oxidizing archaeon from a municipal wastewater treatment system.* Nature Publishing Group, 2017: p. 1-16.

12. Stratton, F.E. and P.L. McCarty, *Prediction of nitrification effects on the dissolved oxygen balance of streams.* Current Research, 1967. **1**(5): p. 405-410.

13. Randall, A.C.W., et al., *Nitrite build-up in activated sludge resulting from temperature effects.* 1984. **56**(9): p. 1039-1044.

14. Kitzinger, K., et al., *Characterization of the first “Candidatus nitrotoga” isolate reveals metabolic versatility and separate evolution of widespread nitrite-oxidizing bacteria.* mBio, 2018. **9**(4): p. 1-16.

15. Blackburne, R., et al., *Kinetic characterisation of an enriched Nitrospira culture with comparison to Nitrobacter.* Water Research, 2007. **41**(14): p. 3033-3042.

16. Vadivelu, V.M., et al., *Stoichiometric and Kinetic Characterisation of Nitrobacter in Mixed Culture by Decoupling the Growth and Energy Generation Processes.* Biotechnology and Bioengineering, 2006. **94**(6): p. 1176-1188.

17. Lafuente, J., J.A. Baeza, and I. Jubany, *Start-up of a nitrification system with automatic control to treat highly concentrated ammonium wastewater : Experimental results and modeling.* 2008. **144**: p. 407-419.

18. Hao, X., J.J. Heijnen, and M.C.M.V. Loosdrecht, *Model-based evaluation of temperature and inflow variations on a partial nitrification – ANAMMOX biofilm process.* 2002. **36**: p. 4839-4849.

19. Henze, M., et al., *Activated sludge models ASM1, ASM2, ASM2 and ASM3*. 2002. 121-121.

20. Station, R.E., F.A. Skinner, and N. Walker, *Growth of Nitrosomonas europaea in batch and continuous culture.* 1961. **349**: p. 339-349.

21. Belser, L.W. and E.L. Schmidt, *Growth and oxidation kinetics of three genera of ammonia oxidizing.* 1980. **7**: p. 213-216.

22. Keen, G.A. and J.I. Prosser, *Steady state and transient growth of autotrophic nitrifying bacteria.* 1987. **147**: p. 73-79.

23. Stehr, G., et al., *The ammonia-oxidizing nitrifying population of the River Elbe estuary.* 1995. **17**: p. 177-186.

24. French, E., et al., *Ecophysiological characterization of ammonia-oxidizing archaea and bacteria from freshwater.* Applied and Environmental Microbiology, 2012. **78**(16): p. 5773-5780.

25. Glover, H.E., *The relationship between inorganic nitrogen oxidation and organic carbon production i batch and chemostat cultures of marine nitrifying bacteria.* Archives of Microbiology, 1985. **142**: p. 45-50.

26. Kits, K.D., et al., *Kinetic analysis of a complete nitrifier reveals an oligotrophic lifestyle.* Nature Publishing Group, 2017. **549**(7671): p. 269-272.

27. Park, B.-j., et al., *Cultivation of Autotrophic Ammonia-Oxidizing Archaea from Marine Sediments in Coculture with Sulfur-Oxidizing Bacteria Cultivation of Autotrophic Ammonia-Oxidizing Archaea from Marine Sediments in Coculture with Sulfur-Oxidizing Bacteria ᰔ †.* 2010(March 2014).

28. Santoro, A.E. and K.L. Casciotti, *Enrichment and characterization of ammonia- oxidizing archaea from the open ocean : phylogeny , physiology and stable isotope fractionation.* The ISME Journal, 2011. **5**(11): p. 1796-1808.

29. Qin, W., et al., *Influence of Oxygen Availability on the Activities of Ammonia-oxidizing Influence of oxygen availability on the activities of ammonia-oxidizing archaea.* 2017(February).

30. Könneke, M., et al., *Isolation of an autotrophic ammonia-oxidizing marine archaeon.* Nature, 2005. **437**(7058): p. 543-546.

31. Martens-habbena, W., et al., *Ammonia oxidation kinetics determine niche separation of nitrifying Archaea and Bacteria.* 2009. **461**(October).

32. Bayer, B., et al., *Physiological and genomic characterization of two novel marine thaumarchaeal strains indicates niche differentiation.* 2015. **10**(5): p. 1051-1063.

33. Bock, E., et al., *A new facultatively nitrite oxidizing bacterium, Nitrobacter vulgaris sp. nov.* 1990. **153**: p. 105-110.

34. Nowka, B., H. Daims, and E. Spieck, *Comparison of Oxidation Kinetics of Nitrite-Oxidizing Bacteria : Nitrite Availability as a Key Factor in Niche Differentiation.* Applied and Environmental Microbiology, 2015. **81**(2): p. 745-753.

35. Ushiki, N., et al., *Nitrite oxidation kinetics of two Nitrospira strains : The quest for competition and ecological niche differentiation.* 2017. **123**(5).

36. Hunik, J.H., H.J.G. Meijer, and J. Tramper, *Kinetics of Nitrobacter agilis at extreme substrate, product and salt concentrations.* Applied Microbiology and Biotechnology, 1993. **40**(2-3): p. 442-448.

37. Both, G.J., S. Gerards, and H.J. Laanbroek, *Kinetics of nitrite oxidation in two Nitrobacter species grown in nitrite-limited chemostats.* Archives of Microbiology, 1992. **157**: p. 436-441.

38. Gay, G., A. Corman, and L.D. Biologic, *Comparative Study of the Growth of Two Strains of Nitrobacter in Batch and Continuous Culture.* 1984: p. 99-105.

39. Kindaichi, T., et al., *Population Dynamics and In Situ Kinetics of Nitrifying Bacteria in Autotrophic Nitrifying Biofilms as Determined by Real-Time Quantitative PCR.* Biotechnology and Bioengineering, 2006. **94**: p. 1111-1121.

40. Watson, S.W. and J.B. Waterbury, *Characteristics of two marine nitrite oxidizing bacteria, Nitrospina gracilis nov. gen. nov. sp. and Nitrococcus mobilis nov. gen. nov. sp.* Archives of Microbiology, 1971. **77**: p. 203-230.

41. Ishii, K., et al., *Enrichment and Physiological Characterization of a Cold-Adapted Nitrite-Oxidizing Nitrotoga sp. from an Eelgrass Sediment.* 2017. **83**(14): p. 1-14.

42. Kitzinger, K., et al., *Single cell analyses reveal contrasting life strategies of the two main nitrifiers in the ocean.* Nature Communications, 2020. **11**(1): p. 1-12.

43. Spieck, E., et al., *Characterization of a new marine nitrite oxidizing bacterium , Nitrospina watsonii sp . nov ., a member of the newly proposed phylum “ Nitrospinae ” ଝ.* Systematic and Applied Microbiology, 2014. **37**(3): p. 170-176.

44. Keuter, S., *Characterization of nitrifying bacteria in marine recirculation aquaculture systems with regard to process optimization.* P.h.D work, 2011: p. 1-114.

45. Sánchez, O., et al., *Nitrification rates in a saline medium at different dissolved oxygen concentrations.* Biotechnology Letters, 2001. **23**: p. 1597-1602.

46. Laanbroek, H.J., P.L.E. Bodelier, and S. Gerards, *Oxygen consumption kinetics of Nitrosomonas europaea and Nitrobacter hamburgensis grown in mixed continuous cultures at different oxygen concentrations.* Archives of Microbiology, 1994. **161**: p. 156-162.

47. Laanbroek, H.J. and S. Gerards, *Competition for limiting amounts of oxygen between grown in mixed continuous cultures.* 1993: p. 453-459.

48. González-Cabaleiro, R., T.P. Curtis, and I.D. Ofiţeru, *Bioenergetics analysis of ammonia-oxidizing bacteria and the estimation of their maximum growth yield.* Water Research, 2019. **154**: p. 238-245.

49. Button, D.K., *Nutrient Uptake by Microorganisms according to Kinetic Parameters from Theory as Related to Cytoarchitecture.* 1998. **62**(3): p. 636-645.

50. Yoshioka, T., H. Terai, and Y. Saijo, *Growth kinetic studies of nitrifying bacteria by th eimmunoflurescent counting method.* J. Gen. Appl. Microbiol., 1982. **28**: p. 169-180.

51. Ward, B.B., *Kinetic studies on ammonia and methane oxidation by Nitrosocccus oceanus.* Archives of Microbiology, 1987. **147**: p. 126-133.

52. Sakoula, D., et al., *Enrichment and physiological characterization of a novel comammox Nitrospira indicates ammonium inhibition of complete nitrification.* The ISME Journal, 2020.

53. Tsai, Y.-l. and O.H. Tuovinen, *Oxygen uptake activity by Nitrobacter spp . in the presence of metal ions and sulfooxyanions.* 1985. **28**: p. 11-14.

54. Jacob, J., et al., *Oxidation kinetics and inverse isotope effect of marine nitrite-oxidizing isolates.* 2017. **80**: p. 289-300.

55. Sharma, B. and R.C. Ahler, *Nitrification and nitrogen removal.* Water Research, 1976. **11**(1): p. 897-925.

56. Bruijn, P.D., et al., *Growth of Nitrosomonas europaea on hydroxylamine.* 1995. **125**: p. 179-184.

57. Könneke, M., et al., *Ammonia-oxidizing archaea use the most energy- efficient aerobic pathway for CO2 fixation.* Proceedings of the National Academy of Sciences of the United States of America, 2014. **111**(22).

58. Li, F., et al., *Genome ‑ scale metabolic model analysis indicates low energy production efficiency in marine ammonia ‑ oxidizing archaea.* AMB Express, 2018. **2**: p. 0-11.

59. Hunik, J.H., et al., *Co-immobilized Nitrosomonas europaea and Nitrobacter agilis cells: validation of a dynamic model for simultaneous substrate conversion and growth in K-carrageenan gel beads.* Biotechnology and Bioengineering, 1994. **43**: p. 1153-1163.

60. Ehrich, S., et al., *A new obligately chemolithoautotrophic , nitrite-oxidizing bacterium , Nitrospira moscoviensis sp . nov . and its phylogenetic relationship.* 1995: p. 16-23.

61. Starkenburg, S.R., D.J. Arp, and P.J. Bottomley, *D-Lactate metabolism and the obligate requirement for CO2 during growth on nitrite by the facultative lithoautotroph Nitrobacter hamburgensis.* Microbiology, 2008. **154**: p. 2473-2481.

62. Watson, S.W., et al., *Nitrospira marina gen. nov. sp. nov.: a chemolithotrophic nitrite-oxidizing bacterium.* 1986(144): p. 1-7.

63. Whittaker, M., et al., *Electron transfer during the oxidation of ammonia by the chemolithotrophic bacterium Nitrosomonas europaea.* Biochimica et Biophysica Acta - Bioenergetics, 2000. **1459**(2-3): p. 346-355.

64. Sedlacek, C.J., et al., *A Physiological and Genomic Comparison of Nitrosomonas Cluster 6a and 7 Ammonia-Oxidizing Bacteria.* Microbial Ecology, 2019. **78**(4): p. 985-994.

65. Thandar, S.M., et al., *Ecophysiology and comparative genomics of nitrosomonas mobilis ms1 isolated from autotrophic nitrifying granules of wastewater treatment bioreactor.* Frontiers in Microbiology, 2016. **7**(NOV): p. 1-14.

66. Schäfer, G. and H. Penefsky, *Bioenergetics. Energy conservation and conversion*. Vol. 53. 2008: Springer. 312-312.

67. Walker, C.B., et al., *Nitrosopumilus maritimus genome reveals unique mechanisms for nitrification and autotrophy in globally distributed marine crenarchaea.* Proceedings of the National Academy of Sciences of the United States of America, 2010. **107**(19): p. 8818-8823.

68. Starkenburg, S.R., et al., *Genome sequence of the chemolithoautotrophic nitrite-oxidizing bacterium Nitrobacter winogradskyi Nb-255.* Microbiology, 2006. **72**(3): p. 2050-2063.

69. Nomoto, T., Y. Fukumori, and T. Yamanakat, *Membrane-Bound Cytochrome c Is an Alternative Electron Donor for Cytochrome aa3 in Nitrobacter winogradskyi.* American society for Microbiology, 1993. **175**(14): p. 4400-4404.

70. Tanaka, Y., Y. Fukumori, and T. Yamanaka, *Purification of cytochrome a1c1 from Nitrobacter agilis and characterization of nitrite oxidation system of the bacterium.* Archives of Microbiology, 1983. **135**: p. 265-271.

71. Lücker, S., et al., *A Nitrospira metagenome illuminates the physiology and evolution of globally important nitrite-oxidizing bacteria.* Proceedings of the National Academy of Sciences of the United States of America, 2010. **107**(30): p. 13479-13484.

72. Lücker, S., et al., *The genome of Nitrospina gracilis illuminates the metabolism and evolution of the major marine nitrite oxidizer.* Frontiers in Microbiology, 2013. **4**(February): p. 1-19.

73. Arai, H., et al., *Enzymatic characterization and in vivo function of five terminal oxidases in Pseudomonas aeruginosa.* Journal of Bacteriology, 2014. **196**(24): p. 4206-4215.

74. Cooper, C.E., et al., *Nitric oxide and peroxynitrite cause irreversible increases in the K m for oxygen of mitochondrial cytochrome oxidase: In vitro and in vivo studies.* Biochimica et Biophysica Acta - Bioenergetics, 2003. **1607**(1): p. 27-34.

75. Garcia-Horsman, J.A., B. Barquera, and J.E. Escamilla, *Two different aa3‐type cytochromes can be purified from the bacterium Bacillus cereus.* European Journal of Biochemistry, 1991. **199**(3): p. 761-768.

76. Preisig, O., et al., *A high-affinity cbb3-type cytochrome oxidase terminates the symbiosis-specific respiratory chain of Bradyrhizobium japonicum.* Journal of Bacteriology, 1996. **178**(6): p. 1532-1538.

77. Kita, K., K. Konishi, and Y. Anraku, *Terminal Oxidases of Escherichia coli Aerobic Respiratory Chain.* J. Biol. Chem., 1984. **259**(5): p. 3368-3374.

78. Kolonay, J.F., et al., *Purification and characterization of the cytochrome bd complex from Azotobacter vinelandii: Comparison to the complex from Escherichia coli.* Journal of Bacteriology, 1994. **176**(13): p. 4177-4181.

79. Mason, M.G., et al., *Cytochrome bd confers nitric oxide resistance to Escherichia coli.* Nature Chemical Biology, 2009. **5**(2): p. 94-96.

80. Belevich, I., et al., *Oxygenated complex of cytochrome bd from Escherichia coli: Stability and photolability.* FEBS Letters, 2005. **579**(21): p. 4567-4570.

81. Belevich, I., et al., *Cytochrome bd from Azotobacter vinelandii: Evidence for high-affinity oxygen binding.* Biochemistry, 2007. **46**(39): p. 11177-11184.

82. D'Mello, R., S. Hill, and R.K. Poole, *Determination of the oxygen affinities of terminal oxidases in Azotobacter vinelandii using the deoxygenation of oxyleghaemoglobin and oxymyoglobin: Cytochrome bd is a low-affinity oxidase.* Microbiology, 1994. **140**(6): p. 1395-1402.

83. Smith, A., S. Hill, and C. Anthony, *The purification, characterization and role of the d-type cytochrome oxidase of Klebsiella pneumoniae during nitrogen fixation.* Journal of General Microbiology, 1990. **136**(1): p. 171-180.

84. D'Mello, R., S. Hill, and R.K. Poole, *The cytochrome bd quinol oxidase in Escherichia coli has an extremely high oxygen affinity and two oxygen-binding haems: Implications for regulation of activity in vivo by oxygen inhibition.* Microbiology, 1996. **142**(4): p. 755-763.

85. Rice, C.W. and W.P. Hempfling, *Oxygen-linmited continuous culture and respiratory energy conservation in Escherichia coli.* Journal of bacteriology, 1978. **134**(1): p. 115-124.

86. Hirai, T., et al., *Expression of multiple cbb3 cytochrome c oxidase isoforms by combinations of multiple isosubunits in Pseudomonas aeruginosa.* Proceedings of the National Academy of Sciences of the United States of America, 2016. **113**(45): p. 12815-12819.

87. Bergersen, F.J. and G.L. Turner, *Properties of terminal oxidase systems of bacteroids from root nodules of soybean and cowpea and of N2-fixing bacteria grown in continuous culture.* Journal of General Microbiology, 1980. **118**(1): p. 235-252.

88. Ohgaki, S. and C. Wantawin, *Nitrification*. 1989. 247-267.

89. Wiesmann, U., *Biological Nitrogen Removal from Wastewater.* 1994. **51**.

90. Blackburne, R. and Æ.Z. Yuan, *Partial nitrification to nitrite using low dissolved oxygen concentration as the main selection factor.* 2008: p. 303-312.

91. Boon, B. and H. Laudelout, *Kinetics of the nitrite oxidation by Nitrobacter winogradskyi.* Journal of bacteriology, 1960. **79**: p. 39-42.

92. Park, M.-r., H. Park, and K. Chandran, *Molecular and Kinetic Characterization of Planktonic Nitrospira spp. Selectively Enriched from Activated Sludge.* 2017.

93. Bristow, L.A., et al., *Ammonium and nitrite oxidation at nanomolar oxygen concentrations in oxygen minimum zone waters.* 2016. **113**(38).

94. Li, Y., et al., *A novel ammonia-oxidizing archaeon from wastewater treatment plant : Its enrichment , physiological and genomic characteristics.* Nature Publishing Group, 2016(March): p. 1-11.

95. Antoniou, P., et al., *Effect of temperature and ph on the effective maximum specific growth rate of nitrifying bacteria.* Water Research, 1990. **24**(1): p. 97-101.

96. Kim, J.G., et al., *Cultivation of a highly enriched ammonia-oxidizing archaeon of thaumarchaeotal group I.1b from an agricultural soil.* Environmental Microbiology, 2012. **14**(6): p. 1528-1543.

97. Anthonisen, A.C., et al., *Inhibition of nitrification by ammonia and nitrous acid.* Journal of the Water Pollution Control Federation, 1976. **48**(5): p. 835-852.

98. Blackburne, R., et al., *Determination of Growth Rate and Yield of Nitrifying Bacteria by Measuring Carbon Dioxide Uptake Rate.* Water Environment Research, 2007. **79**(12): p. 2437-2445.

99. Zhang, Y., et al., *Optimization of the medium for the growth of Nitrobacter winogradskyi by statistical method.* Letters in Applied Microbiology, 2018. **67**(3): p. 306-313.
